# Intragenic suppressors unravel the role of the SCREAM ACT-like domain for bHLH partner selectivity in stomatal development

**DOI:** 10.1073/pnas.2117774119

**Published:** 2022-02-16

**Authors:** Hyemin Seo, Krishna Mohan Sepuru, Aarthi Putarjunan, Lyndsey Aguirre, Benjamin A. Burrows, Keiko U. Torii

**Affiliations:** ^a^HHMI, The University of Texas at Austin, Austin, TX 78712;; ^b^Department of Molecular Biosciences, The University of Texas at Austin, Austin, TX 78712;; ^c^HHMI, University of Washington, Seattle, WA 98195;; ^d^Department of Biology, University of Washington, Seattle, WA 98195

**Keywords:** stomatal development, cell-type differentiation, bHLH proteins, heterodimer, ACT-like domain

## Abstract

Multicellular organisms develop specialized cell types to achieve complex functions of tissues and organs. The basic helix–loop–helix (bHLH) proteins act as master regulatory transcription factors of such specialized cell types. Plant stomata are cellular valves in the aerial epidermis for efficient gas exchange and water control. Stomatal differentiation is governed by sequential actions of three lineage-specific bHLH proteins, SPEECHLESS (SPCH), MUTE, and FAMA, specifying initiation and proliferation, commitment, and terminal differentiation, respectively. A broadly expressed bHLH, SCREAM (SCRM), heterodimerizes with SPCH/MUTE/FAMA and drives stomatal differentiation via switching its partners. Yet nothing is known about its heterodimerization properties or partner preference. Here, we report the role of the SCRM C-terminal ACT-like (ACTL) domain for heterodimerization selectivity. Our intragenic suppressor screen of a dominant *scrm-D* mutant identified the ACTL domain as a mutation hotspot. Removal of this domain or loss of its structural integrity abolishes heterodimerization with MUTE, but not with SPCH or FAMA, and selectively abrogates the MUTE direct target gene expression. Consequently, the *scrm-D ACTL* mutants confer massive clusters of arrested stomatal precursor cells that cannot commit to differentiation when redundancy is removed. Structural and biophysical studies further show that SPCH, MUTE, and FAMA also possess the C-terminal ACTL domain, and that ACTL•ACTL heterodimerization is sufficient for partner selectivity. Our work elucidates a role for the SCRM ACTL domain in the MUTE-governed proliferation–differentiation switch and suggests mechanistic insight into the biological function of the ACTL domain, a module uniquely associated with plant bHLH proteins, as a heterodimeric partner selectivity interface.

## Significance

Whether animal neurons or plant cellular valves (called stomata), specialized cell-type differentiation is directed by the lineage-specific basic helix–loop–helix (bHLH) transcription factors that typically form heterodimers with ubiquitous bHLH proteins. How does a broadly expressed bHLH protein switch its lineage-specific heterodimeric partners? Here we identify a structural module, called the ACT-like domain, in the plant bHLH protein SCREAM. This domain plays a role in partner bHLH selectivity and is critical for the proliferation-to-differentiation switch within the cell lineages to make stomata, plant cellular valves for gas exchange and water control. Our work provides mechanistic insight into how plant transcription factors control cell-fate specification through an unanticipated heterodimeric partner selectivity interface.

Multicellular organisms develop diverse tissue types with specialized cells that perform unique, essential functions. Initiation of progenitor cells and a switch to commitment and differentiation are the defining events of cell-type differentiation, whereby precise spatiotemporal control of transcription factors underlies functional tissue patterning. Basic-helix–loop–helix (bHLH) proteins are prevalent, conserved transcription factors regulating specialized cell-type differentiation in eukaryotes, whether metazoan neurogenesis and myogenesis or plant stomatal differentiation (1–5).

Development of stomata, adjustable valves on the aerial land plant epidermis for optimal gas exchange and water loss, is specified by sequential actions of three stomatal-lineage bHLH transcription factors, SPEECHLESS (SPCH), MUTE, and FAMA. Each of these sister bHLHs is expressed in a specific developmental time window and drives initiation, commitment, and terminal differentiation of stomatal cell lineages. SPCH triggers the entry into stomatal cell lineages from the protodermal state and promotes subsequent asymmetric cell divisions of an early stomatal-lineage precursor called a meristemoid. The meristemoid reiterates asymmetric divisions, each time producing a new meristemoid and its sister cell known as a stomatal-lineage ground cell (SLGC). Subsequently, MUTE commits to the differentiation of a meristemoid to a guard mother cell (GMC) and orchestrates the single symmetric division of the GMC. Finally, FAMA promotes and maintains the terminal differentiation of mature guard cells, in which each pair of guard cells surrounds a pore (6–8). The *spch*, *mute*, and *fama* loss-of-function mutants exhibit an epidermis devoid of any stomatal lineages (*spch*), with arrested meristemoids (*mute*), and aberrant GMCs with excessive symmetric cell divisions (*fama*) (6–8). Conversely, their overexpression confers an epidermis with excessive meristemoids (*SPCH* overexpression), solely composed of stomata (*MUTE* overexpression), and singular guard cells (*FAMA* overexpression), respectively (6–8). Therefore, these bHLHs are both necessary and sufficient for the specification of cell state and are stomatal lineage–specific master regulatory transcription factors.

It is well established that bHLH proteins bind DNA and function as obligatory dimers via the helix-loop-helix (HLH) domain (1, 9) and SPCH/MUTE/FAMA are no exceptions. A previous study identified SCREAM (SCRM, also known as ICE1) and its partially redundant paralog, SCRM2, as heterodimeric partners of SPCH/MUTE/FAMA (10). SCRM and SCRM2 are absolutely required for SPCH/MUTE/FAMA to function: *scrm scrm2* double-knockout mutants exhibit epidermis without any stomatal-lineage cells, identical to *spch*. Likewise, the SCRMs are required for SPCH and MUTE to induce their target gene expression (10, 11). Such targets include cell–cell signaling components, such as *TOO MANY MOUTHS* (*TMM*) receptor–like protein, *EPIDERMAL PATTERNING FACTOR2* (*EPF2*) signaling peptide required for proper stomatal spacing, as well as *SCRM* itself (11–13). In addition, SCRM regulates stomatal differentiation not only as a partner bHLH, but it also functions as a scaffold to bridge the upstream inhibitory cell–cell signal to inhibit SPCH activity. This occurs through SCRM’s mitogen-activated protein kinase (MAPK) anchoring motif, named as a KRAAM motif. The dominant-active *scrm-D* mutation disrupts this KRAAM motif and fails to recruit MAPK to destabilize SPCH protein, resulting in the stomata-only epidermis (14).

As a broadly expressed bHLH protein, SCRM switches partners from SPCH to MUTE to FAMA to integrate the three cell-transitional steps of stomatal differentiation. It is not known, however, if SCRM has any heterodimerization preference over SPCH, MUTE, or FAMA, nor if there is any difference in the heterodimerization properties of SCRM with each of them. To gain insight into the structure–function of SCRM protein in the context of stomatal differentiation, we performed suppressor mutagenesis of its gain-of-function *scrm-D* allele. We identified the C-terminal region that constitutes an ACT-like (ACTL) domain (15) as an intragenic suppressor mutation hotspot. Although the ACTL domain is present in approximately one third of plant bHLH proteins and serves as a dimerization module (15, 16), the in vivo biological significance of this domain remains unclear.

We report here that the SCRM ACTL domain is necessary and sufficient for stomatal bHLH partner specificity. We found that mutations within the SCRM ACTL domain specifically abolish heterodimerization with and abrogate direct target gene expression by MUTE, but not with SPCH or FAMA. Consequently, the *scrm-D* C-terminal ACTL mutants exhibit striking massive clusters of arrested meristemoids in the absence of *SCRM2*. Our structural and biophysical analyses delineate the impact of the SCRM ACTL domain mutation. Finally, we note that SPCH/MUTE/FAMA also possess a C-terminal ACTL domain, and through biophysical and domain-swap experiments, we show that the ACTL domains are sufficient to impact partner selectivity of SCRM with SPCH vs. MUTE. Combined, our work uncovers the critical role of the SCRM ACTL domain for MUTE-governed proliferation–differentiation switch within the stomatal cell lineage, and further provides insight into the biological function of the ACTL domain, an elusive domain prevalent in plant bHLH transcription factors.

## Results

### *scrm-D* Intragenic Suppressor Mutations Fall into the C-Terminal ACTL Domain.

**Fig. 1.**
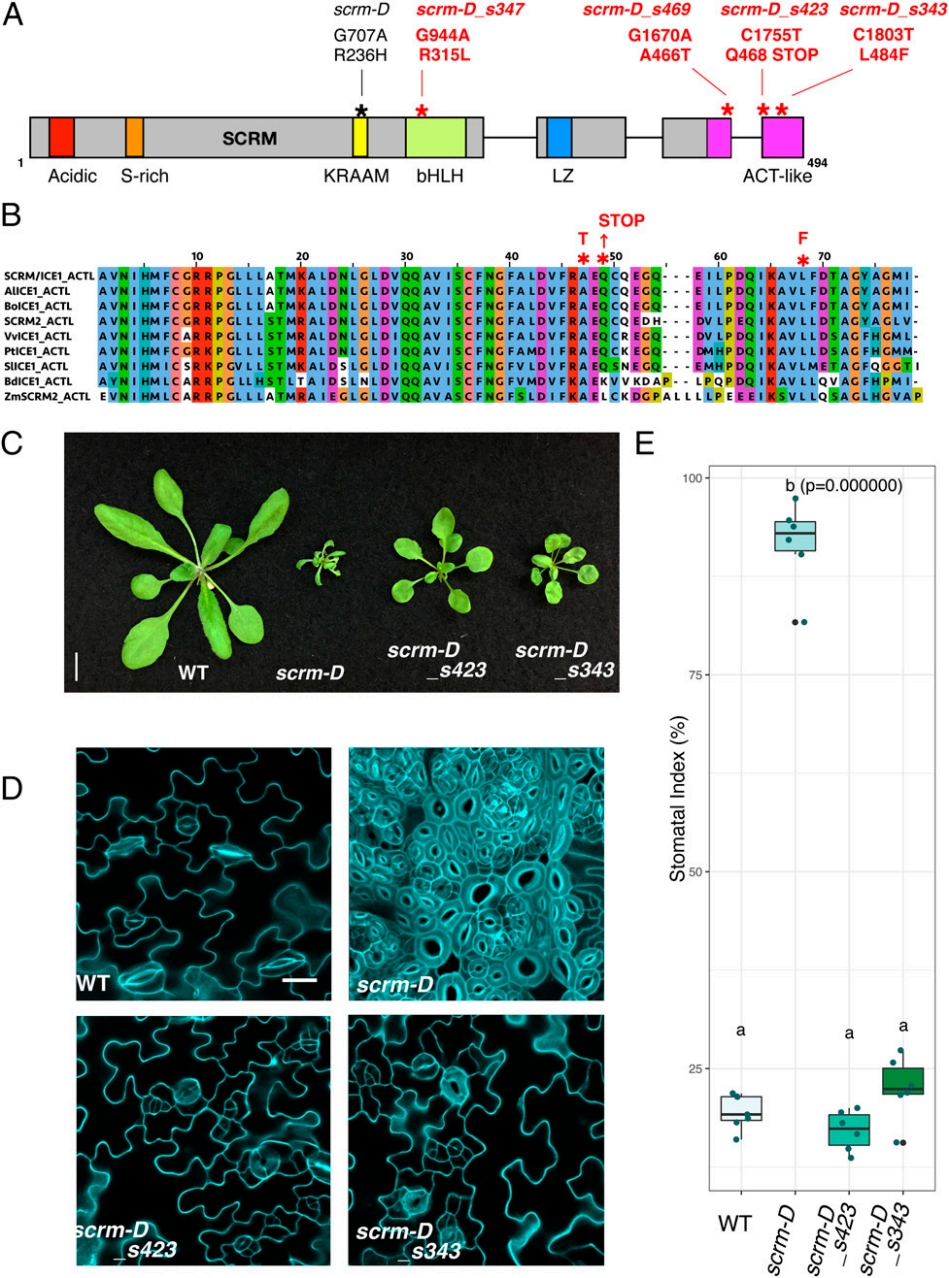
Intragenic suppressors of *scrm-D* highlight the importance of its C-terminal region. (*A*) SCRM domains and location of mutations. S-rich, serine rich; LZ, leucine zipper. The original *scrm-D* mutation is in black; newly isolated suppressor mutations are in red. Both DNA substitutions and their effects in translated protein sequence are indicated. (*B*) Amino acid sequence alignment of the ACTL domain from SCRM and representative orthologs and paralogs. ClustalW was used to generate the alignment. Red asterisks, the site of suppressor mutations and the consequences of mutations. *Al*, *Arabidopsis lyrata*; *Bo*, *Brassica oleracea*; *Vv, Vitis vinifera*; *Pt*, *Populus trichocarpa*; *Sl*, *Solanum lycospersicum*; *Bd*, *Brachypodium distachyon*; *Zm*, *Zea mays*; T, threonine; F, phenylalanine*.* (*C*) Mature plant phenotype. Shown are 3-wk-old plants of wild-type (WT), *scrm-D*, *scrm-D_s423*, and *scrm-D_s343*. The suppressors partially restore growth defects. (Scale bar, 10 mm.) (*D*) Cotyledon abaxial epidermis from 7-d-old seedlings of WT, *scrm-D*, *scrm-D_s423*, and *scrmD-s343*. Images are taken under the same magnification. (Scale bar, 20 μm.) (*E*) Quantitative analysis of stomatal index. *n* = 6 for each genotype. One-way ANOVA followed by Tukey’s honestly significant difference (HSD) analysis was performed. *scrm-D* (group b) is significantly different from all others (*P* = 0.000000), whereas the *scrm-D* suppressors are not significantly different from WT (group a).

To unravel how SCRM regulates stomatal differentiation, we performed a suppressor mutagenesis screen of the gain-of-function *scrm-D* allele by using ethyl methanesulfonate (EMS) (details in *Materials and Methods*). Among the five suppressors we isolated, one (suppressor line 63) corresponds to a new missense allele of *SPCH*, a known interacting partner of SCRM in early stomatal-lineage cells (6, 7, 10, 11). The remaining four suppressor lines, 343, 347, 423, and 469, all harbored a second site mutation within the *SCRM* open-reading frame (At3G26744); thus, they are *scrm-D* intragenic suppressors. Accordingly, these alleles (C1803T, G944A, C1755T, and G1670A) were named *scrm-D_s343*, *scrm-D_s347*, *scrm-D_s423*, and *scrm-D_s469*, respectively (Fig. 1*A*).

The SCRM protein contains multiple domains: an N-terminal acidic domain, a serine-rich region, a KRAAM MAP kinase docking motif, a bHLH, and a leucine-zipper domain (10, 14, 17) (Fig. 1*A*). The *scrm-D_s347* mutation replaces one of the arginine residues within the basic region that serves as a DNA-binding interface to leucine (R315L) (Fig. 1*A* and *SI Appendix*, Fig. S1), presumably abrogating the DNA-binding capacity of the scrm-D protein. This corroborates our previous finding that the DNA binding is required for the constitutive stomatal differentiation by *scrm-D* (10). In addition, the SCRM protein possesses a C-terminal conserved domain with similarity to a bacterial ACT domain (named after three of the proteins that contain it: aspartate kinase, chorismate mutase, and TyrA) (18) (Fig. 1*B*). This domain was described for the Maize R bHLH protein as an ACTL domain (15, 16, 19). Interestingly, the molecular lesions in the three *scrm-D* suppressor alleles fell into the C-terminal ACTL domain: an alanine-to-threonine substitution at the second to the last amino acid of exon 3 (A466T), a leucine-to-phenylalanine substitution (L484F) within the last exon, and a nonsense mutation (Q468STOP) that truncates the entire ACTL domain (Fig. 1 *A* and *B*).

We further characterized the two suppressor alleles with mutations in exon 4: *scrm-D_s423* and *scrm-D_s343*. Both suppressor lines increased overall plant size compared to *scrm-D* (Fig. 1*C*). They both rescued the *scrm-D* all-stomata phenotype, giving rise to a cotyledon epidermis without stomatal clustering and a stomatal index statistically indistinguishable from the wild type (Fig. 1 *D* and *E*). A nearly normal stomatal phenotype was also observed in the epidermis from other photosynthetic organs of these suppressors, including stems, rosette leaves, cauline leaves, and sepals (*SI Appendix*, Fig. S2). These results suggest that the SCRM C terminus is required for *scrm-D*’s ability to trigger stomatal differentiation.

### SCRM ACTL Domain Is Required for the MUTE-Governed Proliferation–Differentiation Switch.

**Fig. 2.**
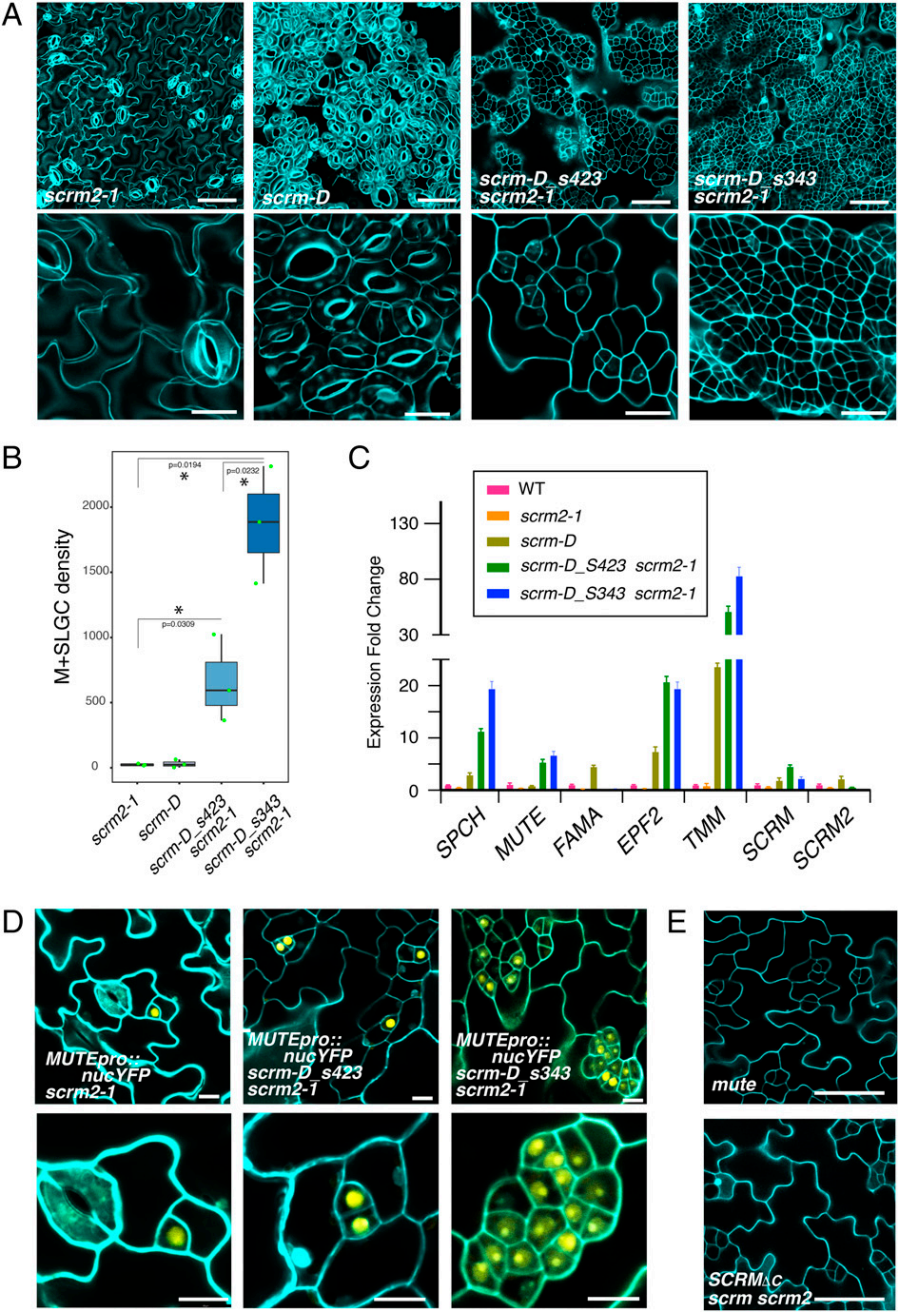
*scrm-D* C-terminal mutations confer arrested meristemoids when genetic redundancy is unmasked. (*A*) Abaxial cotyledon epidermis from 10-d-old *Arabidopsis* seedlings of (from *Left* to *Right*) *scrm2-1*, *scrm-D*, *scrm-D_s423 scrm2-1*, and *scrm-D_s343 scrm2-1. Top*: Low magnification. (Scale bars, 50 μm.) *Bottom*: Higher magnification. (Scale bars, 20 μm.) Massive clusters of arrested meristemoids are observed in the *scrm-D* suppressor alleles in the absence of *SCRM2* (*Right Two Panels*). (*B*) Quantitative analysis of meristemoid and SLGC (M+SLGC) density. *n* = 3. *t* test was performed for a pairwise comparison of suppressor alleles in the *scrm2-1* background and control *scrm2-1* genotype, as well as between *scrm-D_s423 scrm2-1* and *scrm-D_s343 scrm2-1.* *P < 0.05. (*C*) qRT-PCR analysis of core stomatal genes in wild-type (WT), *scrm2-1*, *scrm-D*, *scrm-D_s423 scrm2-1*, and *scrm-D_s343 scrm2-1.* Expression was normalized against actin (*ACT2*), and expression fold change was normalized against the WT values. Three biological replicates were performed. For each biological replicate, three technical replicates were performed. (*D*) Reporter *MUTEpro::nucYFP* expression in *scrm-D* C-terminal mutants in the absence of *SCRM2*. (*E*) Phenotypic resemblance of *mute* (*Top*) and *SCRMpro::SCRM_ΔC_* in *scrm scrm2* background (*Bottom*). Both mutants give rise to arrested meristemoids. Also see Fig. 5*D*.

It is known that SCRM2, an SCRM paralog, functions redundantly with SCRM. The *scrm* single loss-of-function mutant alone confers a penetrant, weak defect in stomatal development, and the severe *spch*-like epidermis (that is devoid of stomatal cell lineages) is observed only when both *SCRM* and *SCRM2* genes are lost (10). Therefore, to address whether the remaining functional *SCRM2* may mask the effects of the *scrm-D* intragenic suppressors, we introduced *scrm-D_s423* and *scrm-D_s343* into the *scrm-2* knockout mutant background. Strikingly, both *scrm-D_s423 scrm2* and *scrm-D_s343 scrm2* double mutants conferred an epidermis with numerous clusters of arrested meristemoids that are not able to differentiate into stomata even at 10 d after germination (Fig. 2*A*). Quantitative analyses corroborate phenotypic observations that meristemoids are dramatically increased in these suppressor lines in the absence of *SCRM2* (Fig. 2*B*). The meristemoid clustering phenotype of *scrm-D_s343 scrm2* is more severe than in *scrm-D_s423 scrm2* (Fig. 2 *A* and *B*), reflecting its slightly weaker suppression of the *scrm-D* phenotype (Fig. 1).

To molecularly characterize the clustered-meristemoid phenotype, we examined the expression levels of selected stomatal-lineage marker genes by qRT-PCR (Fig. 2*C*). Transcript levels of early stomatal-lineage genes, *SPCH* as well as its direct targets *TMM* and *EPF2*, are highly elevated in *scrm-D_s423 scrm2* and *scrm-D_s343 scrm2* when compared with the wild type, *scrm-2*, and *scrm-D*. Likewise, high expression levels of *MUTE*, a late meristemoid-to-early GMC marker gene, were detected in *scrm-D_s423 scrm2* and *scrm-D_s343 scrm2* (Fig. 2*C*). Consistent with the absence of guard cell differentiation in these mutants, no *FAMA* transcripts were detected in *scrm-D_s343 scrm2* and *scrm-D_s423 scrm2* (Fig. 2*C*).

Both morphological and molecular phenotypes of *scrm-D* suppressors in the *scrm2* background resemble the *scrm-D mute* double mutant, in which nearly all cotyledon/leaf protodermal cells enter the stomatal cell lineage due to enhanced SPCH activity by *scrm-D*, but fail to exit from the proliferative meristemoid state due to the absence of *MUTE* (20). However, *scrm-D_s343 scrm2* and *scrm-D_s423 scrm2* harbor a functional *MUTE* locus. Indeed, we detected strong signals of endogenous *MUTE* transcripts and reporter *MUTEpro::nucYFP* in clusters of arrested meristemoids (Fig. 2 *C* and *D*). Therefore, although the meristemoid in *scrm-D_s343 scrm2* and *scrm-D_s423 scrm2* strongly expresses *MUTE*, it cannot transition into a GMC (and further to stomata). These results suggest that the *scrm-D* ACTL domain mutation compromises posttranscriptional functions of MUTE as a master regulator of stomatal differentiation, but not its expression.

The original *scrm-D* intragenic suppressor alleles retain the R236H substitutions within the KRAAM motif (14). To delineate the specific function of the SCRM ACTL domain in stomatal differentiation, we expressed the wild-type version of SCRM lacking the entire ACTL domain (the identical truncation as that in *scrm-D_s423*) driven by the native *SCRM* promoter (*SCRMpro::SCRM_ΔC_*) in a *scrm scrm2* double-knockout background. Consistent with having a functional MPK3/6-recruiting KRAAM motif, *SCRMpro::SCRM_ΔC_* no longer conferred excessive stomatal-lineage entry. It still developed an epidermis with arrested meristemoids after rounds of asymmetric divisions, a phenotype indistinguishable from *mute* (7) (Fig. 2*E*). On the basis of these findings, we conclude that the SCRM C-terminal ACTL domain is required for the transition from proliferation to differentiation within the stomatal cell lineages, the step governed by MUTE.

### The SCRM C-Terminal ACTL Domain Is Required for Heterodimerization with MUTE, but Not SPCH or FAMA.

**Fig. 3.**
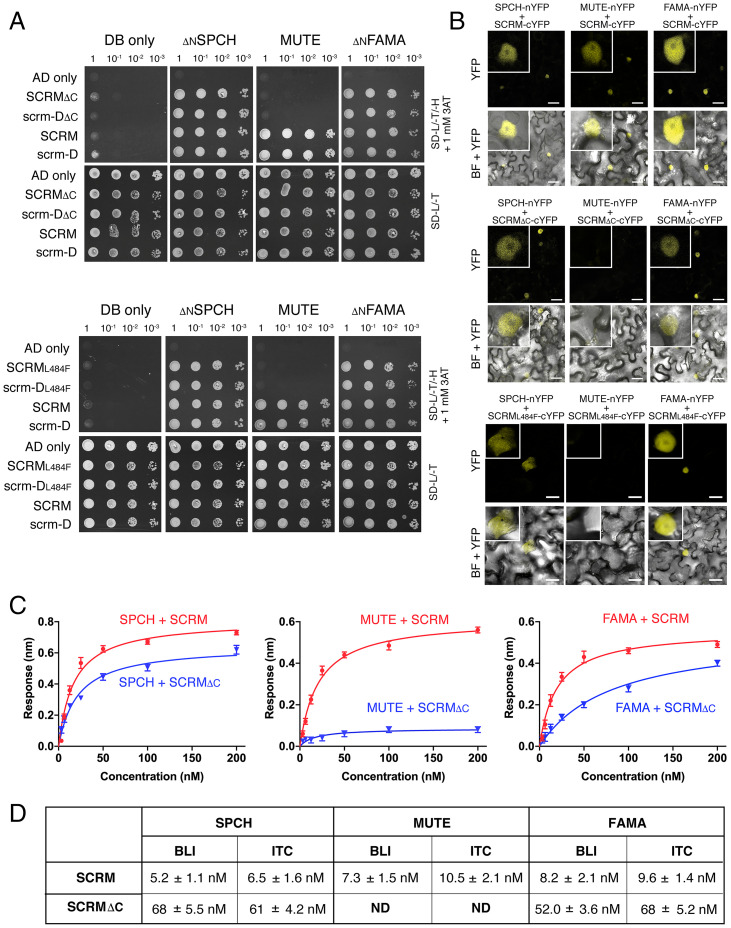
SCRM ACTL mutations selectively abrogate heterodimerization with MUTE but not SPCH or FAMA. (*A*) Y2H analysis. DNA binding domain alone (DB only), _ΔN_SPCH, MUTE, and _ΔN_FAMA were used as bait, and activation domain alone (AD only), SCRM, scrm-D, SCRM_ΔC_, scrm-D_ΔC_, SCRM_L484F_, and scrm-D_L484F_ were used as prey. Yeast clones were spotted in 10-fold serial dilutions on appropriate selection media. Experiments were repeated three times. (*B*) BiFC assays. *N. benthamiana* leaves were infiltrated with pairwise combinations of full-length SPCH-nYFP, MUTE-nYFP, and FAMA-nYFP with SCRM-cYFP (*Top*) as well as with SCRM_ΔC_-cYFP (*Middle*) and with SCRM_L484F_-cYFP (*Bottom*). YFP, confocal imaging of YFP signal; BF, bright field. *Inset*: Magnified image of a representative nucleus. (Scale bar, 25 μm.) (*C*) Quantitative analysis of SCRM/SCRM_△C_ • SPCH/MUTE/FAMA heterodimer interactions by BLI. Shown are in vitro binding response curves for purified SPCH, MUTE, and FAMA with GST-fused SCRM (red) and SCRM_△C_ (blue). SPCH, MUTE, and FAMA proteins at seven different concentrations (200, 100, 50, 25, 12.5, 6.25, and 3.125 nM) are subjected to analysis. Data are mean ± SD, representative of two independent experiments. (*D*) Table of *K_d_* values calculated from the BLI and ITC assays. See *SI Appendix*, Fig. S4 and Table S1 for the ITC data.

Whereas SCRM drives cell-state transition within the stomatal lineages via switching its heterodimeric partner bHLH proteins from SPCH to MUTE to FAMA (10), the *SCRM* C-terminal mutations identified via our *scrm-D* suppressor screen specifically conferred the *mute-like* phenotype in the *scrm2* background. To address if these SCRM C-terminal mutations compromise heterodimerization with MUTE, we employed a series of protein–protein interaction assays (Fig. 3 and *SI Appendix*, Figs. S3 and S4 and Tables S1 and S2). For yeast two-hybrid (Y2H) assays, the N-terminal domain of SPCH and FAMA was removed to prevent their autoactivation in the Y2H system (10, 14). _ΔN_SPCH, MUTE, and _ΔN_FAMA fused to the DNA binding domain were subjected to analysis in pairwise combinations with scrm-D_ΔC_ (corresponds to scrm-D_s423), SCRM_ΔC_, scrm-D_L484F_ (corresponds to scrm-D_s343), and SCRM_L484F_ fused to the activation domain. As expected, _ΔN_SPCH, MUTE, and _ΔN_FAMA all associated with the full-length SCRM and scrm-D. In contrast, only MUTE, but not _ΔN_SPCH or _ΔN_FAMA, failed to interact with the C-terminal mutant versions of SCRM/scrm-D (Fig. 3*A*).

We next performed bimolecular fluorescence complementation (BiFC) assays in planta, which, unlike Y2H, allows the use of full-length versions of SPCH and FAMA (Fig. 3*B*). Indeed, upon coexpression of SCRM_ΔC_-cYFP, scrm-D_ΔC_-cYFP, SCRM_L484F_-cYFP, and scrm-D_L484F_-cYFP with SPCH-nYFP, MUTE-nYFP, and FAMA-nYFP in *Nicotiana benthamiana* leaves, only MUTE lost interaction with SCRM_ΔC_, scrm-D_ΔC_, SCRM_L484F_, and scrm-D_L484F_, whereas both SPCH and FAMA were able to interact with the SCRM (and scrm-D) C-terminal mutant variants (Fig. 3*B* and *SI Appendix*, Fig. S3). The reconstituted YFP signals of SPCH and FAMA with the mutant versions of SCRM/scrm-D appear somewhat weaker (Fig. 3*B* and *SI Appendix*, Fig. S3), implying that, although these C-terminal ACTL mutations in SCRM (and scrm-D) proteins selectively abrogate association with MUTE, they may also generally weaken SCRM heterodimerization capacity or protein stability.

To quantitatively characterize the heterodimerization property of SCRM vs. SCRM_ΔC_ with SPCH/MUTE/FAMA, we expressed and purified recombinant proteins and performed two independent biophysical assays: biolayer interferometry (BLI) and isothermal titration calorimetry (ITC). The BLI assay showed that SCRM binds with SPCH, MUTE, and FAMA with high affinity, with dissociation constant (*K_d_*) values at 5.2 ± 1.1, 7.3 ± 1.5, and 8.2 ± 2.1 nM, respectively, with fast association and slow dissociation kinetics (Fig. 3 *C* and *D* and *SI Appendix*, Table S2). SCRM_ΔC_ interaction with SPCH and FAMA was reduced roughly by 10-fold (*K_d_* values 68 ± 5.5 and 52 ± 3.6 nM for SPCH and FAMA, respectively) (Fig. 3 *C* and *D*), consistent with slightly reduced YFP signals detected in BiFC assays (Fig. 3*B*). In contrast, we detected no binding of SCRM_ΔC_ and MUTE (Fig. 3 *C* and *D*).

The bHLH proteins bind to the target DNA sequence (E-box: CANNTG; CACGTG is canonical) as a dimer. Their basic residues serve as the DNA-binding interface and the adjacent HLH domain facilitates dimerization (1). To address if the presence of target DNA fragments may stabilize the interaction of SCRM_ΔC_ with MUTE, we next performed BLI assays in the presence of a natural target DNA (*TMM* promoter fragment and its mutant E-box version as a negative control) as well as a synthetic E-box fragment (*SI Appendix*, Fig. S5). The DNA fragments had minimal effects on the heterodimerization of SCRM with SPCH/MUTE/FAMA as well as that of SCRM_ΔC_ with SPCH and FAMA (*SI Appendix*, Fig. S5 and Table S2). Interestingly, in the presence of target DNA fragments, we were able to fit the binding of SCRM_ΔC_ with MUTE (*K_d_* values of 565 ± 3.5 nM with the *TMM* promoter and 545 ± 5.5 nM with the synthetic E-box fragment), although the values are 10-fold higher than with SPCH or FAMA (*SI Appendix*, Fig. S4 and Table S2). In contrast, no binding of SCRM_ΔC_ and MUTE was detected in the presence of the control mutant E-box fragment (*SI Appendix*, Fig. S4 and Table S2). These findings suggest that DNA-bound bHLH domains of SCRM_ΔC_ and MUTE aid their association. However, a slight enhancement of this association is not sufficient to drive the differentiation of GMCs from meristemoids in vivo (Fig. 2).

In addition to quantitative binding kinetics, the ITC assays can provide multiple thermodynamic parameters from a single experiment: enthalpy (Δ*H*), entropy (Δ*S*), and free energy (Δ*G*) that is also related to the *K_d_* and stoichiometry of protein–protein interactions (21). The binding isotherm of SCRM to SPCH, SCRM to MUTE, SCRM to FAMA, SCRM_ΔC_ to SPCH, and SCRM_ΔC_ to FAMA fit best to a single binding site model, yielding a stoichiometry of one heterodimer partner per SCRM/SCRM_ΔC_, indicating that SCRM forms a heterodimer with SPCH/MUTE/FAMA at a 1:1 ratio (*SI Appendix*, Fig. S4 and Table S1). The ITC data indicate that the binding of SCRM and SCRM_ΔC_ to their heterodimeric partners is predominantly driven by enthalpic interactions and is entropically disfavored. The *K_d_* values obtained by ITC are essentially identical to those by BLI (Fig. 3*D*). SCRM binds with SPCH, MUTE, and FAMA tightly, with *K_d_* values of 6.5 ± 1.6 nM, 10.5 ± 2.1 nM, and 9.6 ± 1.4 nM, respectively. SCRM_ΔC_ binds to SPCH and FAMA, with *K_d_* values of 61 ± 4.2 nM and 68 ± 5.2 nM, respectively. In accordance with the BLI assays, the binding of SCRM_ΔC_ to SPCH and FAMA was reduced roughly by 10-fold, and again, SCRM_ΔC_ showed negligible binding to MUTE (Fig. 3*D* and *SI Appendix*, Fig. S4).

Collectively, our protein–protein interaction assays in yeast, in planta, and in quantitative biophysical assays provide compelling evidence that the SCRM C-terminal ACTL domain is required for heterodimerization with MUTE, but not with SPCH or FAMA, and suggest a role for the ACTL domain in mediating bHLH partner selectivity.

### The SCRM ACTL Domain Is Required for the Expression of Direct Target Genes of MUTE, but Not SPCH or FAMA.

**Fig. 4.**
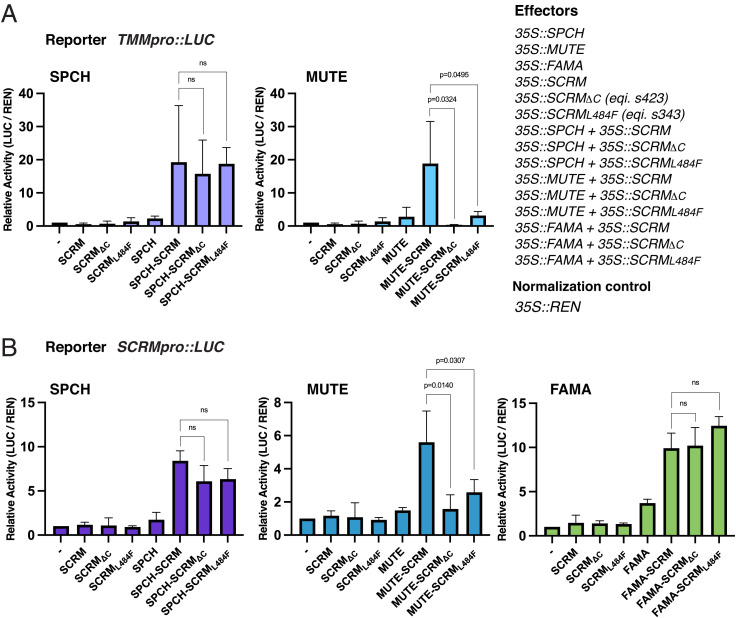
SCRM ACTL domain is required for expression of MUTE target genes but not that of SPCH or FAMA. (*A*) Dual-Luc assays of *TMM* promoter (*TMMpro::LUC*) with individual or pairwise combinations of effector SPCH, MUTE, SCRM, and SCRM C-terminal mutant versions. Relative Luc activity (LUC/REN) was normalized against the empty vector control. One-tailed Student’s *t* test was performed for selected pairwise combinations. Three biological replicates were performed, each with three technical replicates. Error bars, SEM. *Right*: Schematics of dual-Luc assays performed in this study. (*B*) Dual-Luc assays of *SCRM* promoter (*SCRMpro::LUC*) with individual or pairwise combinations of effector SPCH, MUTE, FAMA, SCRM, and SCRM C-terminal mutant versions. LUC/REN was normalized against the empty vector control. Welch’s two-sample *t* test was performed for selected pairwise combinations. Three biological replicates were performed, each with three technical replicates. Error bars, SEM. ns, not significant.

As sister bHLH proteins promoting stomatal differentiation, SPCH and MUTE share overlapping sets of direct target genes (12, 13). We postulated that MUTE is unable to induce transcription of its direct targets in combination with SCRM C-terminal mutants. To address this hypothesis, we performed dual-luciferase (Luc) transactivation assays using *N. benthamiana*. We used reporter Luc constructs driven by the native promoters of *TMM* and *SCRM*, both of which are shared direct targets of SPCH and MUTE (11–13). We also tested if FAMA can transactivate the expression of its likely target, *SCRM*, when heterodimerized with SCRM or its C-terminal mutant. Individual introduction of these bHLH proteins alone did not transactivate Luc activity of *TMMpro::Luc* and *SCRMpro::Luc*, consistent with their heterodimeric mode of action as transcriptional activators (Fig. 4).

As expected, strong Luc reporter activities for both promoters are detected when SPCH, MUTE, or FAMA is coexpressed with SCRM (Fig. 4). Likewise, coexpression of SPCH with either SCRM_ΔC_ or SCRM_L484F_ conferred strong *TMMpro::Lu*c and *SCRMpro::Luc* activities statistically indistinguishable from those of SPCH with full-length SCRM (Fig. 4). Likewise, FAMA coexpressed with SCRM_ΔC_ or SCRM_L484F_ triggered SCRMpro::Luc activities as much as FAMA with full-length SCRM (Fig. 4). In contrast, MUTE coexpressed with SCRM_ΔC_ or SCRM_L484F_ failed to induce *TMM* or *SCRM* reporters, leaving the Luc activity not statistically different from the expression of MUTE alone (Fig. 4). The observed strong transactivation of target genes by SPCH or FAMA with either SCRM_ΔC_ or SCRM_L484F_ indicates that these mutant SCRM proteins are expressed and accumulated sufficiently in planta. Taken together, we conclude that MUTE cannot form a heterodimer with the SCRM C-terminal mutants in vivo to induce the direct target genes required for stomatal differentiation.

### Structural Integrity of SCRM ACTL Underpins the Heterodimerization Specificity.

The in vivo developmental phenotypes, reporter transactivation assays, and protein–protein interaction analyses all support the notion that the C-terminal ACTL domain of SCRM is required for functional heterodimerization with MUTE to drive differentiation of meristemoids to GMCs. The ACTL domain of Maize R has been extensively characterized by in vitro biochemical assays and in Y2H analysis as a homodimerization module (16, 22). To gain insight into the SCRM ACTL domain structure and the impact of the L484F mutation, we employed a structural modeling approach (*Materials and Methods*).

**Fig. 5.**
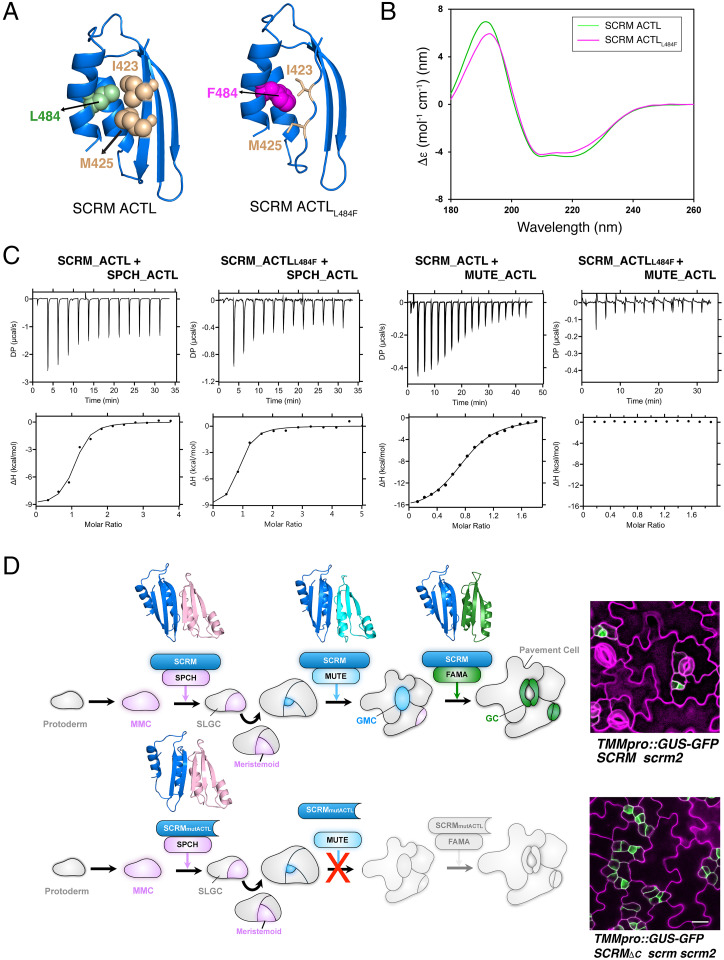
SCRM ACTL domain structure and the impact of the mutation on partner selectivity. (*A*) Structural modeling of SCRM ACTL domain (*Left*), with L484 residue highlighted in green and I423 and M425 residues in sand color. Structural modeling of SCRM ACTL_L484F_ (*Right*) abrogates the intramolecular association due to steric hindrance introduced by the F484 (magenta) residue, predicted to cause a collapse of the third β-sheet. (*B*) CD spectra of SCRM ACTL domain (green) and ACTL_L484F_ (magenta). (*C*) ITC of purified SCRM_ACTL vs. SPCH_ACTL, SCRM_ACTL_L484F_ vs. SPCH_ACTL, SCRM_ACTL vs. MUTE_ACTL, and SCRM_ACTL_L484F_ vs. MUTE_ACTL. Shown are experimental values ± fitting errors. ACTL_L484F_ of SCRM completely abolishes its heterodimerization with MUTE ACTL, whereas it does not change the interaction with SPCH ACTL. For exact *K_d_* values and thermodynamic parameters, see *SI Appendix*, Table S3. (*D*) Model diagram. *Top*: Under normal condition, SCRM (blue) drives cell-state transitional steps via sequentially forming a heterodimer with SPCH (lilac), MUTE (cyan), and FAMA (green). Structural models of ACTL•ACTL heterodimerization between SCRM and SPCH/MUTE/FAMA are presented above each bHLH heterodimer. This leads to proper stomatal development. *Right*: Wild-type cotyledon abaxial epidermis from a *scrm2* seedling expressing a stomatal-lineage marker *TMMpro::GUS-GFP* (green). MMC; meristemoid mother cell, SLGC, stomatal-lineage ground cell; GMC, guard mother cell; GC, guard cell. *Bottom*: The SCRM mutant, either without ACTL domain or with mutant ACTL domain that lost structural integrity (SCRM_mutACTL_), can still heterodimerize with SPCH to drive the initiation and proliferation of stomatal precursor cells. However, SCRM_mutACTL_ cannot form a heterodimer with MUTE. Consequently, meristemoids arrest after rounds of asymmetric cell divisions, leading to an epidermis devoid of stomata. *Right*: Cotyledon abaxial epidermis from a *SCRMpro::SCRM_ΔC_ scrm scrm2* seedling expressing stomatal-lineage marker *TMMpro::GUS-GFP* (green). (Scale bar, 20 μm.) ns, not significant.

Whereas the prototypical bacterial ACT domain exhibits a characteristic arrangement of four β strands and two α helices in a βαββαβ fold (18), the SCRM ACTL domain forms βαββα organization due to its shorter length (Fig. 5 *A*, *Left*). The L484 residue is located within the second α-helix of the βαββα fold and is predicted to act as a key residue to stabilize the tertiary structure via hydrophobic interactions with I423 and M425 residues within the first β-sheet (Fig. 5 *A*, *Left*). The L484F substitution is predicted to cause a collapse of this β-sheet due to steric hindrance by the disruption of hydrophobic interactions that stabilize the compact folding of the ACTL domain (Fig. 5 *A*, *Right*).

Next, to experimentally address the predicted β-sheet collapse, we performed circular dichroism (CD) of purified recombinant SCRM_ACTL and its L484 version (Fig. 5*B*). The CD spectra reveal the presence of α-helices (negative bands at 221 nm and 208 nm) and a β-sheet (positive band at 193 nm) (Fig. 5*B*). Further secondary structure analysis (23) indicates that the SCRM L484F mutation reduces the β-sheet region from 35% to 30% (Fig. 5*C*), which corresponds to the loss of four amino acids within the first β-sheet, thus verifying the modeling prediction. These results suggest that SCRM•MUTE heterodimerization is uniquely sensitive to the structural integrity of the SCRM ACTL domain.

### Partner Selectivity Lies in ACTL•ACTL Heterodimerization.

Unlike Maize R, which functions as a homodimer (15, 16), SCRM drives stomatal differentiation by forming heterodimers with SPCH, MUTE, and FAMA (10). How does the SCRM ACTL domain facilitate interactions with these partner bHLH proteins? Our structural modeling predicts that SPCH, MUTE, and FAMA also possess conserved C-terminal ACTL domains with βαββα folds (*SI Appendix*, Figs. S6 and S7). To gain insight into the ACTL•ACTL heterodimerization, we further generated models for the SCRM_ACTL domain complexed with SPCH_ACTL, MUTE_ACTL, and FAMA_ACTL domains using high ambiguity–driven biomolecular docking (HADDOCK)–based calculations that utilize ambiguous restraints along with shape complementarity and energetics to drive the docking process (*Materials and Methods*). Our modeling revealed that α-helix 1 and β-sheet 2 constitute a heterodimer interface. In all three heterodimer combinations, the interface residues within SCRM are similar, suggesting that the differences in binding strength arise from interface residues within the ACTL domains of SPCH/MUTE/FAMA (*SI Appendix*, Fig. S7). The interface residues in all proteins are a mixture of charge–charge and hydrophobic interactions (*SI Appendix*, Fig. S7). Thus, hydrophobic packing, guided by H-bonding and ionic interactions, likely mediates SCRM-SPCH, SCRM-MUTE, and SCRM-FAMA heterodimer formation via ACTL•ACTL interaction.

To experimentally address whether the ACTL domains of SCRM and its partners are sufficient for heterodimerization, we expressed and purified recombinant ACTL domains from SCRM, SPCH, and MUTE and performed ITC biophysical interaction assays (Fig. 5*C* and *SI Appendix*, Table S3). The SCRM ACTL domain is heterodimerized with the ACTL domains from SPCH and MUTE, with *K_d_* values of 1.2 ± 0.6 μM and 5.1 ± 1.2 μM, respectively. The results indicate that these stomatal bHLH proteins are capable of forming ACTL•ACTL heterodimers, although their interaction strengths are orders of magnitude less than the full-length proteins (∼100 times higher *K_d_* values; Figs. 3*D* and 5*C* and *SI Appendix*, Tables S1 and S2). Next, we examined whether the integrity of the SCRM ACTL domain per se is sufficient to discriminate the ACTL domain of SPCH vs. MUTE. Whereas SCRM ACTL_L484F_ caused no differences in heterodimerization with SPCH ACTL (1.7 ± 0.8 μM), it abolished interaction with MUTE (Fig. 5*C*).

Finally, to examine the specificity of SPCH vs. MUTE ACTL domains in the context of the full-length proteins in planta, we generated chimeric SPCH and MUTE proteins, in which the ACTL domain was domain swapped (*SI Appendix*, Fig. S8 *A* and *B*), and performed dual-Luc transactivation assays on the *TMMpro::LUC* reporter (*SI Appendix*, Fig. S8 *C* and *D*). Strikingly, a chimeric SPCH protein with the MUTE ACTL domain (SPCH_MUTE_ACTL_) diminished its ability to transactivate the reporter when coexpressed with SCRM_ΔC_ and SCRM_L484F_, but not with SCRM (*SI Appendix*, Fig. S8*C*). Conversely, a chimeric MUTE protein with the SPCH ACTL domain (MUTE_SPCH_ACTL_) regained transactivation ability when coexpressed with SCRM_ΔC_ and SCRM_L484F_, just like with SCRM (*SI Appendix*, Fig. S8*D*). Taken together, our results suggest that the SCRM’s partner selectivity lies in ACTL•ACTL heterodimerization and that the ACTL domain within SPCH and MUTE is largely sufficient for this selectivity.

## Discussion

Through genetic, phenotypic, and a series of in vivo and in vitro interaction and reporter assays, we revealed that mutations which remove the SCRM C-terminal ACTL domain or replace its conserved residues (L484F) abrogate its heterodimerization potential with MUTE, but not with SPCH and FAMA (Figs. 3 and 4). Since SCRM directs stomatal cell-state transitions through sequentially forming a heterodimer with SPCH, MUTE, and FAMA (in that order), the stomatal differentiation aborts at the MUTE step, i.e., the transition from proliferation to differentiation in the ACTL domain mutants (Figs. 2 and 5*D*). Through structural modeling and experimental verifications, we uncovered hidden heterodimerization selectivity of SPCH, MUTE, and FAMA with their shared partner SCRM, and further provide insight into the binding properties and biological function of the ACTL domain in plants.

### bHLH ACTL Domain-Buffering Heterodimerization Selectivity.

A third of plant (*Arabidopsis* and maize) bHLH proteins are predicted to possess the C-terminal ACTL domain (15, 19, 22). Extensive in vitro biochemical and Y2H protein–protein interaction assays have been performed on Maize bHLH protein R and related bHLHs to characterize their dimerizations (22). A recent study of a bHLH heterodimeric pair in vascular development, LONESOME HIGHWAY (LHW) and TARGET OF MONOPTEROS 5 (TMO5), revealed that their C-terminal ACTL domain is necessary but not sufficient to trigger root vascular stem cell divisions. Thus, the mode of action and specific functions of these ACTL domains remain unclear (24). Interestingly, homodimerization of the R ACTL domain interferes with the homodimerization of the R bHLH domain as well as prevents subsequent binding to its target DNA sequence, G-box (16). This led to a model whereby the R ACTL domain serves as an inhibitory module that prevents bHLH proteins from forming an active, DNA-binding dimeric configuration (16). Unlike R, however, the presence or absence of SCRM’s ACTL domain affects neither SPCH•SCRM heterodimerization (regardless of the presence or absence of target DNA) nor activation of SPCH direct target gene expression (Figs. 3 and 4 and *SI Appendix*, Figs. S4 and S5). Likewise, *scrm-D_s423* and *scrm-D_s343*, which possess both the constitutively active R236H (*scrm-D*) mutation and the second site mutation that removes or impacts the ACTL domain, triggered excessive asymmetric entry divisions in the *scrm2* background—indicative of SPCH overactivation (Fig. 2). Combined, these findings do not support the role of the SCRM ACTL domain as an autoinhibitory module. Instead, we propose that the SCRM ACTL domain epitomizes an extra safeguard to ensure the robust heterodimerization of stomatal core bHLHs in order to securely drive sequential cell-state transitions (Fig. 5*D*). Indeed, we showed that the isolated SCRM ACTL domain alone can stably heterodimerize with the ACTL domain of SPCH and MUTE (Fig. 5*C*), emphasizing that the ACTL domains are sufficient for stomatal bHLH heterodimerization.

Why is the MUTE•SCRM heterodimer so sensitive to the integrity of the SCRM ACTL domain? SPCH, MUTE, and FAMA also possess the C-terminal ACTL domain with high sequence similarity (*SI Appendix*, Fig. S6), and our structural modeling does not reveal their fundamental differences (*SI Appendix*, Fig. S7*A*). Nevertheless, the isolated SCRM ACTL_L484F_ selectively abolished its interaction with the ACTL domain of MUTE. Different charge distributions among the ACTL intermolecular interaction surfaces (*SI Appendix*, Fig. S7 *B* and *C*) might have a larger role in stable ACTL•ACTL heterodimerization. Strikingly, the domain-swap experiments in planta showed that heterodimerization selectivity between SPCH and MUTE to SCRM largely relies on their ACTL domains (*SI Appendix*, Fig. S8). We thus hypothesize that the C-terminal ACTL domain provides a robust dimerization platform—allowing the bHLH domains to bind to the target genomic sites as heterodimers. The isolated ACTL•ACTL heterodimerization of full-length SCRM-MUTE is five times weaker than that of SCRM-SPCH (*SI Appendix*, Table S2), implying that the ACTL domain offers a somewhat limited platform for the SCRM-MUTE pair. Our finding that the presence of target DNA fragment aids MUTE heterodimerization with SCRM ACTL-domain mutants (*SI Appendix*, Fig. S5 and Table S2) supports this hypothesis. One should be cautious that, while the ACTL domains are sufficient for heterodimerization selectivity, the stomatal bHLH proteins likely execute cell-state transitions via associating with multiple factors. In this regard, it is worth mentioning that SPCH and FAMA possess additional domains that do not exist in MUTE, including an N-terminal extension (SPCH/FAMA), a MAPK substrate domain (SPCH), and a retinoblastoma-like protein binding motif (FAMA), all of which contribute to their specific biological functions (7, 25–27). These additional modules might strengthen the heterodimerization with SCRM during in the in vivo context of stomatal differentiation.

As a versatile partner bHLH, SCRM also forms heterodimers with specialized bHLHs beyond the context of stomatal development. For example, SCRM regulates seed development (more specifically, endosperm breakdown) via interacting with ZHOUPI (ZOU), which belongs to the bHLH Ia (the same phylogenetic clade as SPCH/MUTE/FAMA) (28, 29). Although not originally annotated (28), we find that ZOU also possesses the C-terminal ACTL domain (*SI Appendix*, Figs. S6 and S7*D*). It would be interesting to test the heterodimerization properties of ZOU with SCRM without the proper ACTL domain and address their in vivo ramifications. Such an approach could be expanded to large inventories of the ACTL domain–containing plant bHLHs to collectively survey their partner selectivity.

### Hidden Heterodimerization Selectivity Unmasked by the Loss of the Paralogous Gene.

It has been shown that *SCRM* and *SCRM2* exhibit unequal redundancy, with *SCRM* playing a major role and *SCRM2* being dispensable in the presence of functional *SCRM* (10). We observed that both *scrm-D_s343* and *scrm-D_s423* exhibited a nearly wild-type epidermis with a normal stomatal index (Fig. 1), whereas they confer striking, massive meristemoid clusters in the *scrm2* knockout background (Fig. 2). Thus, the presence of the functional *SCRM2* masks the heterodimerization selectivity and suppresses the excessive stomatal-lineage entry divisions of *scrm-D* with additional lesions in the C-terminal ACTL domain. SCRM2 also possesses the C-terminal ACTL domain that most likely serves as the heterodimerization interface (Fig. 1*B* and *SI Appendix*, Fig. S7). These findings provide a critical implication: at a mechanistic level, the differences in protein–protein interaction affinity can shift the dimerization preference among bHLH proteins. This could in principle be buffered by the presence of a functional paralog retaining a stronger interaction affinity. Reduced activity of such paralogs, however, might unmask the novel heterodimerization selectivity, creating a phenotypic diversity.

An active phenotypic transcriptional compensation—in which the loss of one gene drastically elevates the expression of its paralog—has been proposed as a buffering mechanism underlying tomato stem cell homeostasis (30). Unlike such cases, the loss of *SCRM* does not alter the expression levels of *SCRM2* (10). Evolutionarily, the *SCRM* lineage is well conserved throughout the land plant lineages, from astomous liverwort *Marchantia polymorpha* and moss *Physcomitrella* to grass *Brachypodium distachyon* (14, 31, 32). Analyzing domain structures and heterodimerization properties of SCRM/SCRM2 orthologs with partner SPCH/MUTE/FAMA orthologs may shed light on the role of protein structure–based phenotypic compensation and variations in stomatal patterning in the land plant lineages.

### Similarities and Uniqueness of Lineage-Specific bHLH Heterodimers.

The regulatory logic of how SCRM drives stomatal cell-state transition shows a striking parallel to that of myogenesis and neurogenesis (and other specialized cell-type differentiation) in metazoans, where shared broadly expressed E proteins partner with lineage-specific bHLHs (e.g., MyoD, myogenin, and Myf5 for myogenesis, and Mash1, neuroD, and neurogenin in neurogenesis) to drive lineage specification, precursor proliferation, and differentiation (2, 3, 33, 34). As master regulatory transcription factors, these bHLH proteins have additional protein–protein interaction modules to recruit transcriptional, epigenetic, and signaling machineries (33, 34). For instance, the Hes family bHLH proteins possess a conserved tetrapeptide motif at the C terminus that recruits a transcription corepressor complex (35, 36). However, the C-terminal regions of these metazoan bHLHs do not accompany a domain with defined topology (*SI Appendix*, Fig. S9). The prevalent yet unique family of ACTL domain–containing plant bHLH proteins might implicate co-opted protein modules for transcription factor control of cell-type differentiation in a plant-specific way. Further structure–function studies may clarify such mechanisms.

## Materials and Methods

### Plant Materials.

The *Arabidopsis thaliana* Columbia (Col) accession was used as a wild type. Mutants and transgenic lines used in this study are in the Col background unless otherwise specified. The following plant materials were reported previously: *scrm-D*, *scrm (ice1-2)*, and *scrm2-1* (10); *mute-2* (37); *TMM:GUS-GFP* (38); and *MUTEpro::nucYFP* (39). *scrm-D* suppressors were derived from EMS-mutagenized *scrm-D* (discussed next). Seedlings and plants were grown as previously described (14).

### Mutagenesis and *scrm-D* Suppressor Screen.

Homozygous *scrm-D* seeds were treated with 3% (vol/vol) EMS (Sigma) solution overnight in a fume hood. M2 (second generation of mutagenized) seeds were harvested from individual M1 lines. A total of ∼40,000 M2 seedlings (∼80 seedlings per M1 line) were sown on Murashige and Skoog medium (MS-0) plates and visually screened for the epidermal phenotype 10 to 14 d after germination. Those seedlings exhibiting normal/nearly normal stomatal patterning were genotyped for the presence of the original *scrm-D* mutation (G1004A of At3G26744.1) to eliminate true revertants or wild-type contaminants. Subsequently, the entire coding region of *scrm-D* was sequenced to identify intragenic suppressor mutations. One M2 line (line 67) did not possess any secondary site mutation within the *scrm-D* locus. Since *spch* is epistatic to *scrm-D* (10), the entire *SPCH* open reading frame was subsequently sequenced. The alleles of interest were outcrossed to wild-type Col-0 three times to clean up additional mutations. For primer DNA sequences, see *SI Appendix*, Dataset S1.

### Plasmid Construction and Generation of Transgenic Plants.

For all plasmid constructs generated and used in this study, see *SI Appendix*, Dataset S2. For generating transgenic plants, constructs were transformed into *Agrobacterium* GV3101, and transgenic plants were subsequently generated using the floral dipping method. At least 20 T1 (first generation of transgenic) lines were characterized for the transgenic phenotypes and progenies of those lines segregating monogenic inheritance are used for further studies.

### qRT-PCR.

RNA isolation, complementary DNA (cDNA) preparation, and qRT-PCR analyses were performed as described previously (39). Briefly, RNA was isolated from 10-d-old seedlings using the RNeasy Plant Mini Kit (Qiagen). RNA (1 mg) was converted to cDNA using the iScript cDNA synthesis kit (Bio-Rad). The qPCR reaction was run using iTaq Universal SYBR Green Supermix on the CFX96 real-time system (Bio-Rad). Relative expression was calculated by dividing *ACT2* gene expression over the expression of a gene of interest, and expression fold change for each gene of interest was normalized against the expression in wild-type seedlings. Three biological replicates were performed, and three technical replicates were performed for each biological replicate. For a list of primers, see *SI Appendix*, Dataset S1.

### Y2H Analysis.

Bait and prey constructs were cotransformed into yeast strain AH109 using a yeast transformation kit (Frozen-EZ Yeast Transformation II Kit, Zymo Research). Y2H assays were done using the Matchmaker 3 system (Clontech). The resulting transformants with appropriate positive and negative controls were spotted on synthetically defined (SD) medium (-Leu/-Trp) plates to check for growth in the absence of selection. The transformants were then spotted on SD (-Trp -Leu -His) selection medium containing 0.1 mM, 0.5 mM, and 1 mM 3-amino-1,2,4-triazole (Sigma, A8056). The positive interactors were then scored based on the stringency of the selection.

### BiFC Analysis.

BiFC assays were carried out as described previously (40) with minor modifications. Split YFP constructs were generated for SPCH, MUTE, FAMA, SCRM, scrm-D, SCRM_ΔC_, scrm-D_ΔC_, SCRM_L484F_, and scrm-D_L484F_ by cloning them into either pSPYNE, which contains the N terminus of EYFP protein (nYFP-174 amino acid), or pSPYCE, which contains the C terminus of EYFP protein (cYFP-64 amino acid) (40). See *SI Appendix*, Dataset S1 for detailed information on the constructs. The constructs were transformed into *Agrobacterium tumefaciens* strain GV3101. Bacterial cultures were spun down at 4,500 rpm for 10 min and resuspended in infiltration buffer (10 mM MgCl_2_, 10 mM 2-(N-morpholino)ethanesulfonic acid [pH 5.6], and 150 μM acetosyringone). Bacterial culture densities were adjusted to a final optical density (OD_600_) of 1.0, and the cell suspensions were incubated at room temperature for 4 h prior to infiltration. Equal volumes of cultures carrying the corresponding complementary pair of BiFC constructs (YFPn and YFPc) along with silencing suppressor plasmid, p19 (a gift from Sir David Baulcombe [University of Cambridge, United Kingdom]) (41), were then coinfiltrated into 3- to 4-wk-old *N. benthamiana* leaves. The infiltrated leaves were imaged 2 d postinfiltration using confocal microscopy as described next .

### Confocal Microscopy and Image Analysis.

The *Arabidopsis* epidermis was observed using a Zeiss LSM700, Leica SP5 WLL, or Leica Stellaris 8 inverted confocal microscope. Cell peripheries were visualized by propidium iodide (Molecular Probes) using the following settings: excitation, 515 nm, and emission, 623 to 642 nm. Confocal imaging for *N. benthamiana* leaves was done using the Leica SP5-WLL confocal microscope simultaneously capturing YFP (excitation at 518 nm and emission at 540 nm for EYFP) and bright field differential interference contrast channels. The confocal images were uniformly and linearly adjusted using Adobe Photoshop.

### Dual-Luc Assays.

The reporter Luc constructs pCS001 (pGREEN-0800-LUC), pCS003 (pGREEN-800-*TMMpro*LUC), and pRJH68 (pGREEN-800-SCRM*pro*LUC) and the effector constructs pLJP152 (35Spro::SPCH), pLJP151 (35Spro::MUTE), and pMK165 (35Spro::SCRM) were published previously (10, 11, 42). The reporter and effector constructs were transformed into 5-wk-old *N. benthamiana* leaves via agroinfiltration as previously described (11). Six days after infiltration, tobacco leaves were harvested and assayed using the Dual-Glo Luciferase Assay System kit (Promega, E2920). The tobacco leaves were snap-frozen by liquid N_2_, powdered, and subsequently mixed with 75 μL of the passive lysis buffer provided in the kit. The cellular debris was pelleted by centrifugation at 8,000 × *g* for 1 min. The supernatant was transferred into a well of a white flat-bottom Costar 96-well plate (Corning). An equal volume of Dual-Glo Reagent was added to each well. First, *firefly* Luc activity was measured. A volume of Dual-Glo Stop & Glo Reagent equal to the original tissue lysate was added to each well, and *Renilla* Luc activity was subsequently measured. The assay was performed using a GloMax 96-microplate luminometer (Promega).

### Recombinant Protein Expression and Purification.

SCRM (1-494), SCRM1ΔC (1-404), SCRM ACTL (405-494), SCRM-ACTL_L485F_ (405-494), _ΔN_SPCH (98-364), SPCH-ACTL (285-364), MUTE (1-202), MUTE-ACTL (114-202), and _ΔN_FAMA were cloned into the pGEX-4T-1 vector with an N-terminal glutathione S-transferase (GST) tag and a thrombin cleavage sequence, and MUTE (1-202) was cloned into the pET28a vector with an N-terminal His tag. See *SI Appendix*, Dataset S1 for plasmid information. For protein expression, the constructs were transformed into *Escherichia coli* strain BL21. For each transformant, a single clone was selected and incubated in 5 mL Luria broth (LB) liquid medium with appropriate antibiotics. The overnight-incubated *E. coli* suspensions were transferred to 1 L LB medium and incubated at 37 °C for around 2 h until the OD_600_ reached 0.4 to 0.6. Isopropyl β-D-1-thiogalactopyranoside (0.25 μM final concentration) was added to the cultures, and the strains were incubated at 25 °C for a further 16 h. Cells were harvested by centrifugation at 6,000 rpm and resuspended in lysis buffer (20 mM Tris, 200 mM NaCl, pH 8.0) containing a protease inhibitor mixture tablet (Roche Diagnostic). GST-fused proteins were purified using glutathione agarose resin (Cytiva Sweden AB), and MUTE HIS-tag protein was purified using nickel-nitrilotriacetic acid (Ni-NTA) agarose resin. The soluble portion of the cell lysate was loaded onto a GST-Sepharose column (Cytiva Sweden AB). Nonspecifically bound proteins were removed by washing the column with 20 mM Tris (pH 8.0) and 200 mM NaCl. The bound GST-fused protein was eluted with 10 mM glutathione, 20 mM Tris (pH 8.0), and 200 mM NaCl (pH 8.0). The GST-fused proteins were exchanged with phosphate-buffered saline (PBS) buffer, and then the solution was treated with 50 μg thrombin for 10 to 12 h at 16 °C. The GST portion of the protein was cleaved during thrombin digestion, and then the whole solution was reloaded onto the glutathione *S*-transferase column to obtain pure protein. The purified proteins were further purified by gel filtration on a Superdex-200 column (GE) using fast protein liquid chromatography (Bio-Rad) and phosphate buffer (pH 7.2) as the eluent. Likewise, MUTE-HIS protein was purified using an Ni-NTA column (QIAGEN) followed by gel filtration on a Superdex-200 column (GE). The purity of the protein was checked by SDS-PAGE (*SI Appendix*, Fig. S10).

### ITC.

Binding of SCRM to MUTE, SPCH, FAMA and SCRM_ΔC_ to MUTE, SPCH, FAMA was characterized at 25 °C using a Malvern PEAQ-ITC microcalorimeter (Malvern Panalytical). All protein samples were dialyzed overnight using PBS buffer. Titrations were performed by injecting 1 × 0.5-μl and 12 × 3-μl aliquots of 40 μM SCRM/SCRM_ΔC_ to 4 μM SPCH/MUTE/FAMA in PBS buffer, pH 7.4. Titrations were performed by injecting 1 × 0.5-μl and 17 × 2-μl aliquots of 150 μM SCRM ACTL/SCRM L484F ACTL to 15 μM SPCH ACTL/MUTE ACTL in PBS buffer, pH 7.4. All titrations were carried out at least twice. The raw data were corrected using buffer and protein controls and analyzed using the software supplied by the manufacturer.

### BLI.

The binding affinities of the SPCH, MUTE, and FAMA proteins with GST-tagged SCRM and SCRM_ΔC_ were measured in the presence and absence of the target DNA (*SI Appendix*, Fig. S5) using the Octet Red96 system (ForteBio, Pall Life Sciences) following the manufacturer’s protocols. The optical probes coated with anti-GST were first loaded with 500 nM GST SCRM and SCRM_ΔC_ before kinetic binding analyses. The experiment was performed in 96-well plates maintained at 30 °C. Each well was loaded with 200 μL reaction volume for the experiment. The binding buffer used in these experiments contained 1× PBS supplemented with 0.02% Tween 20. The concentrations of the SPCH/MUTE/FAMA as the analyte in the binding buffer were 200 nM, 100 nM, 50 nM, 25 nM, 12.5 nM, 6.25 nM, and 3.12 nM. To check the SCRM and SCRM_ΔC_ interactions with SPCH/MUTE/FAMA in the presence of the DNA fragments, samples were incubated for 30 min with a 1:4 molar ratio of DNA fragments before performing the experiment. All preformed complexes remained stable as suggested by the constant signal during the washing step after loading. There was no binding of the analytes to the unloaded probes as shown by the control wells. Binding kinetics to all seven concentrations of the analytes were measured simultaneously using default parameters on the instrument. The data were analyzed using the Octet data analysis software. The association and dissociation curves were fit with the 1:1 homogeneous ligand model. The kobs (observed rate constant) values were used to calculate *K_d_*, with steady-state analysis of the direct binding.

### CD.

All CD spectra were collected on a Jasco J-815 Circular Dichroism Spectrometer at 25 °C in PBS buffer, pH 7.2. Samples of SCRM ACTL and ACTL_L485F_ were extensively dialyzed against the buffer and then filtered before determination of protein concentration. Spectra were recorded from 250 to 180 nm with a scan rate of 5 nm·min^−1^ in a 0.1-cm path length cuvette. The final spectral measurements representing an average of five independent scans were corrected for buffer contribution. The secondary structures of the wild-type and mutant ACTL domains were predicted according to Micsonai et al. (23).

### Protein Sequence Alignment and Structural Modeling.

Multiple sequence alignment/sequence conservation analysis was performed using JALVIEW (version 2.8) (43). SCRM, SCRM L485F, SPCH, MUTE, and FAMA ACTL domain homology models were constructed with MODELER (44). This platform builds protein models from query sequences using the solved crystal structures contained at the Research Collaboratory for Structural Bioinformatics Protein Data Bank as templates. The modeled structures were subjected to constrained energy minimization to allow the global energy minimization and structural analysis using the AMBER 12 suite and VADAR (45). Assessment of the stereochemistry of three dimensional models was performed using a Ramachandran plot (PROCHECK) (46), Protein Quality Predictor (ProQ) (47), MolProbity (48) and Protein Structure Analysis (ProSA) (49). Molecular docking of SCRM to the SPCH, MUTE, and FAMA monomers was carried out using the HADDOCK approach (50, 51). The MODELER-generated and AMBER-minimized structures were used for docking. Ambiguous interaction restraints were selected based on reported ACT domain dimer structures (52, 53). The pairwise “ligand interface Rmsd matrix” over all structures was calculated, and the final structures were clustered using an Rmsd cutoff value of 3.5 Å for both SCRM and SPCH/MUTE/FAMA. The clusters were then prioritized using Rmsd and the HADDOCK score (weighted sum of a combination of energy terms).

## Supplementary Material

Supplementary File

## Data Availability

All study data are included in the article and/or supporting information.
