# Supplementary material for: Intragenic suppressors unravel the role of the SCREAM ACT-like domain for bHLH partner selectivity in stomatal development
Source: Proc Natl Acad Sci U S A. 2022 Feb 16;119(9):e2117774119. doi: 10.1073/pnas.2117774119 (PMC8892516; doi:10.1073/pnas.2117774119)
Supplement: Supplementary File [file pnas.2117774119.sapp.pdf]

www.pnas.org

Supplementary Information for

Intragenic suppressors unravel the role of SCREAM ACT-like domain for bHLH partner selectivity in stomatal development

Hyemin Seo, Krishna Mohan Sepuru, Aarthi Putarjunan, Lyndsey Aguirre, Benjamin A. Burrows, Keiko U. Torii

Email: ktorii@utexas.edu

This PDF file includes:

Supplementary Figures and Figure Legends

Fig. S1. The basic region of the *scrm-D* bHLH domain is required for 'stomata-only' epidermis

Fig. S2, Stomatal phenotypes of *scrm-D* intragenic suppressors in diverse aerial epidermis

Fig. S3. BiFC assays showing selective abrogation of heterodimerization between scrm-D ACTL mutants and MUTE

Fig. S4. Thermodynamics of SCRM/SCRM$_{\Delta C}$-SPCH/MUTE/FAMA heterodimer interactions

Fig. S5. Quantitative analysis of SCRM/SCRM$_{\Delta C}$ • SPCH/MUTE/FAMA heterodimer interactions in presence of DNA fragments by BLI.

Fig. S6. SPCH/MUTE/FAMA orthologs possess highly-conserved C-terminal ACTL domains

Fig. S7. Modelled ACTL domain structures and surface charge maps

Fig. S8. Domain-swap experiments show that the SPCH ACTL domain is sufficient to induce target gene expression *in planta* with SCRM ACTL mutants

Fig. S9. Unique structural feature of plant developmental bHLH proteins

Fig. S10. SDS-PAGE gel images of purified recombinant proteins used for biophysical assays

Supplementary Tables

Table S1. Thermodynamic parameters for the binding of SCRM / SCRM$_{\Delta C}$ to SPCH, MUTE, and FAMA

Table S2. Kinetic- and binding constants for SCRM and SCRMΔC to SPCH, MUTE and FAMA using BLI

Table S3. Thermodynamic parameters for the binding of the SCRM / SCRM$_{L484F}$ ACTL domains to SPCH and MUTE ACTL domains

Supplementary Dataset

Dataset S1. List of oligo DNA used in this study (provided as Excel Spreadsheets)

Dataset S2. List of plasmids used in this study (provided as Excel Spreadsheets)

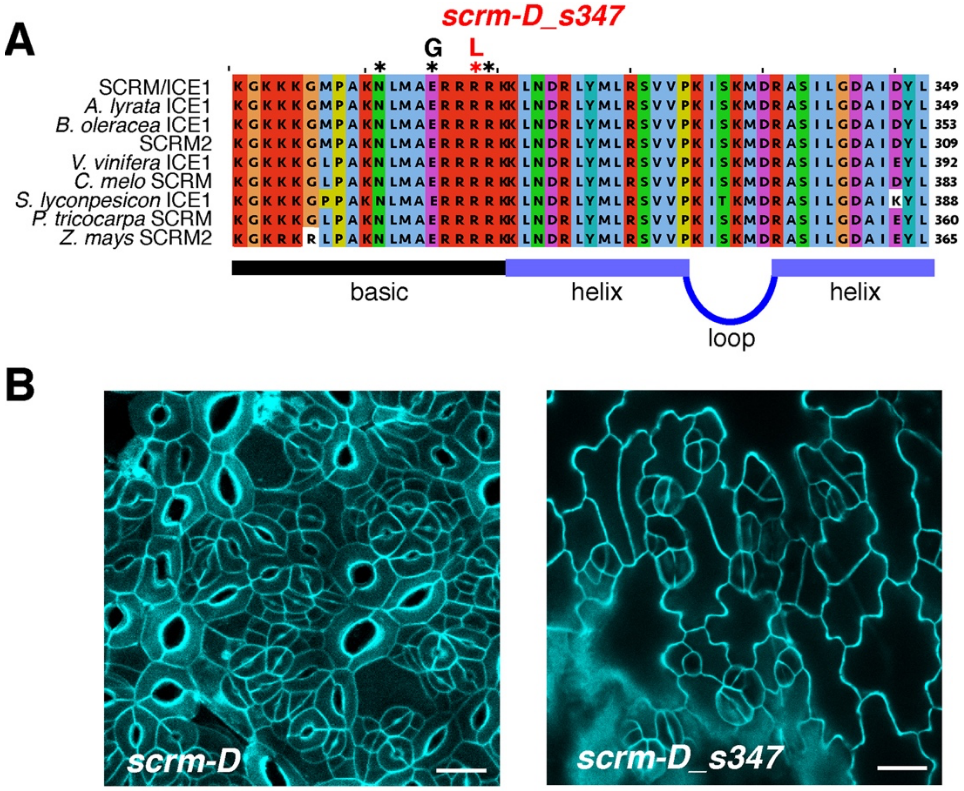

Fig. S1. The basic region of the *scrm-D* bHLH domain is required for 'stomata-only' epidermis

(A) Amino-acid sequence alignment of bHLH domain from SCRM orthologs and paralogs. ClustalW was used to generate the alignment. Core invariant amino-acids (N, E, R) for DNA-binding interface. Red asterisk, the mutation site (R315L) in *scrm-D_s347*; Black asterisks, a core amino acid in which site-directed mutagenesis (E352G) is known to abolish the *scrm-D* phenotype, as demonstrated by Kanaoka *et al.* (2008). Bottom, location of basic (black bar), helix (blue bar), loop (navy blue loop), helix (blue bar) domain.

(B) Confocal microscopy of from the abaxial cotyledon epidermis from 7-day-old seedlings of *scrm-D* (left) and *scrm-D_s347* (right). Scale bars, 20 μm.

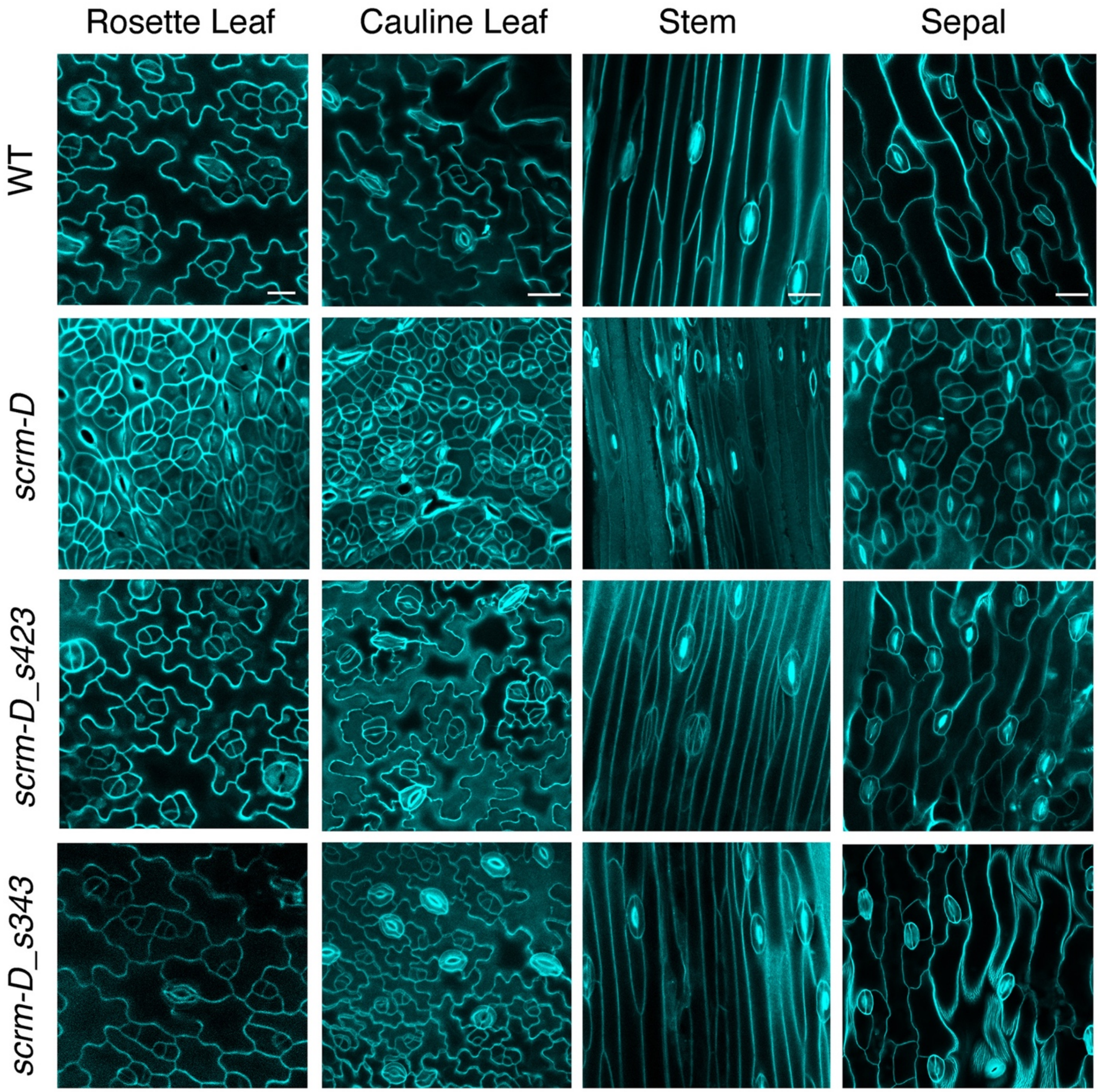

Fig. S2. Stomatal phenotypes of *scrm-D* intragenic suppressors in diverse aerial epidermis

Shown are confocal microscopy images from (left to right) rosette leaf, cauline leaf, stem, and sepal epidermis from 4-week-old (top to bottom) wild-type (WT), *scrm-D*, *scrm-D_s423*, and *scrm-D_s343* plants. Scale bars, 10 μm for rosette leaf and 20 μm for the rest of the organs.

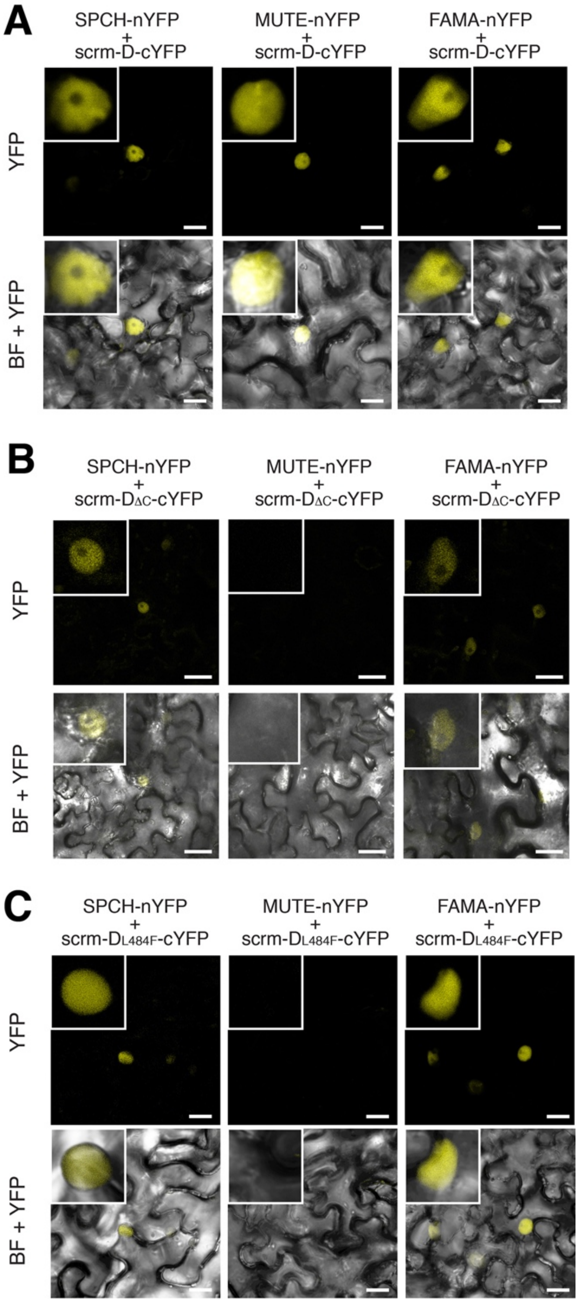

Fig. S3. BiFC assays showing selective abrogation of heterodimerization between scrm-D ACTL mutants and MUTE

Shown are representative BiFC assays of all remaining combinations not shown in Figure 3B. *N. benthamiana* leaves were infiltrated with the following pairwise combinations:

(A) B. SPCH-nYFP, MUTE-nYFP, FAMA-nYFP with scrm-D-cYFP

(B) SPCH-nYFP, MUTE-nYFP, FAMA-nYFP with scrm-D$_{\Delta C}$-cYFP

(C) SPCH-nYFP, MUTE-nYFP, FAMA-nYFP with scrm-D$_{L484F}$-cYFP

YFP, confocal imaging of YFP signal; BF, bright field. Inset, magnified image of a representative nucleus. Scale bars, 25 μm.

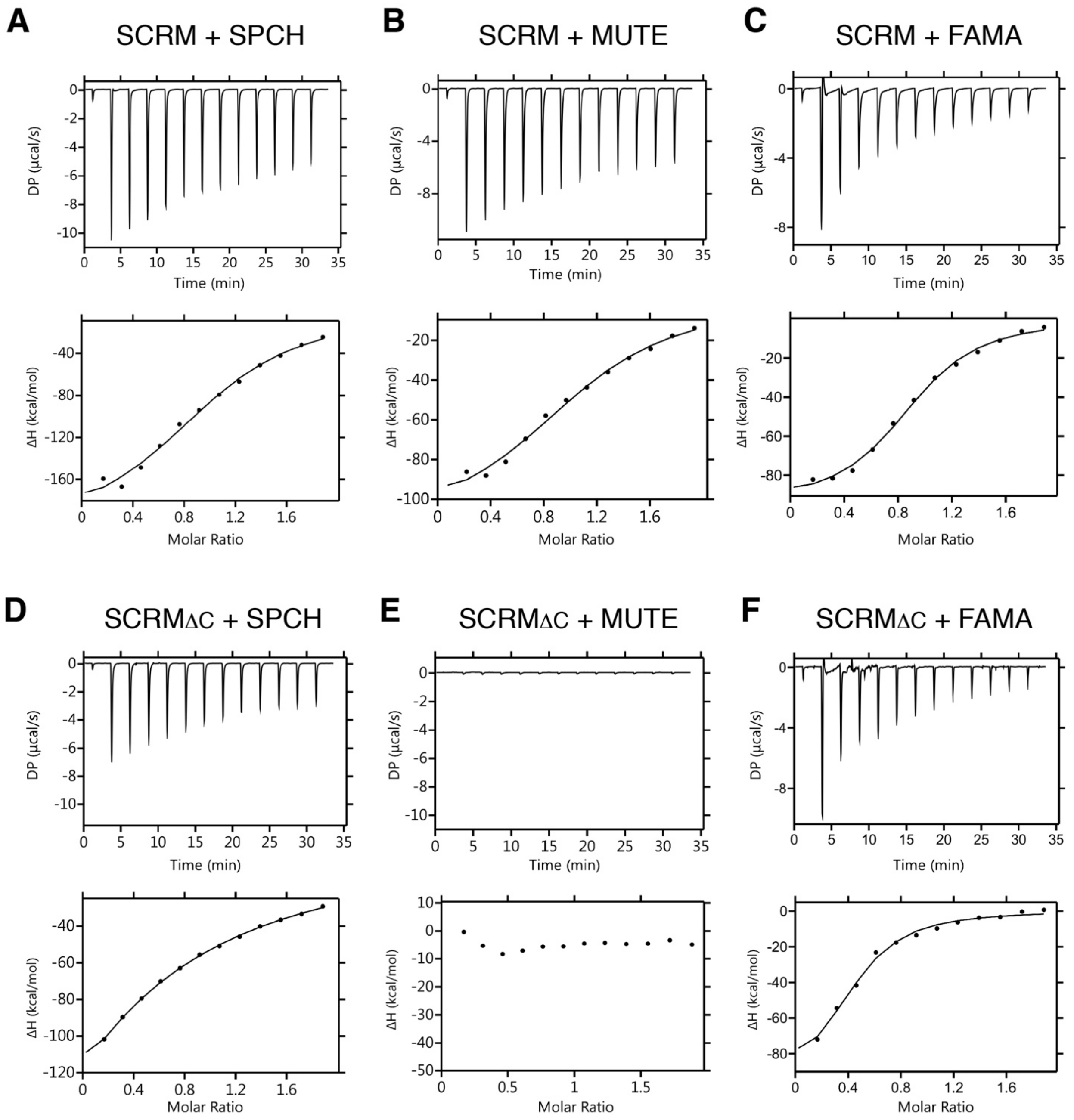

Fig. S4. Thermodynamics of SCRM/SCRM$_{\Delta C}$-SPCH/MUTE/FAMA heterodimer interactions Shown are isotherms corresponding to the binding of SCRM to SPCH (**A**), SCRM to MUTE (**B**), SCRM to FAMA (**C**), SCRM$_{\Delta C}$ to SPCH (**D**), SCRM$_{\Delta C}$ to MUTE (**E**), and SCRM$_{\Delta C}$ to FAMA (**F**). The titrations and the integrated data obtained after subtracting the heat of dilution are shown in the upper and lower panels, respectively. The titrations were performed in the phosphate saline buffer, pH 7.2, at 25 °C. See Methods for details.

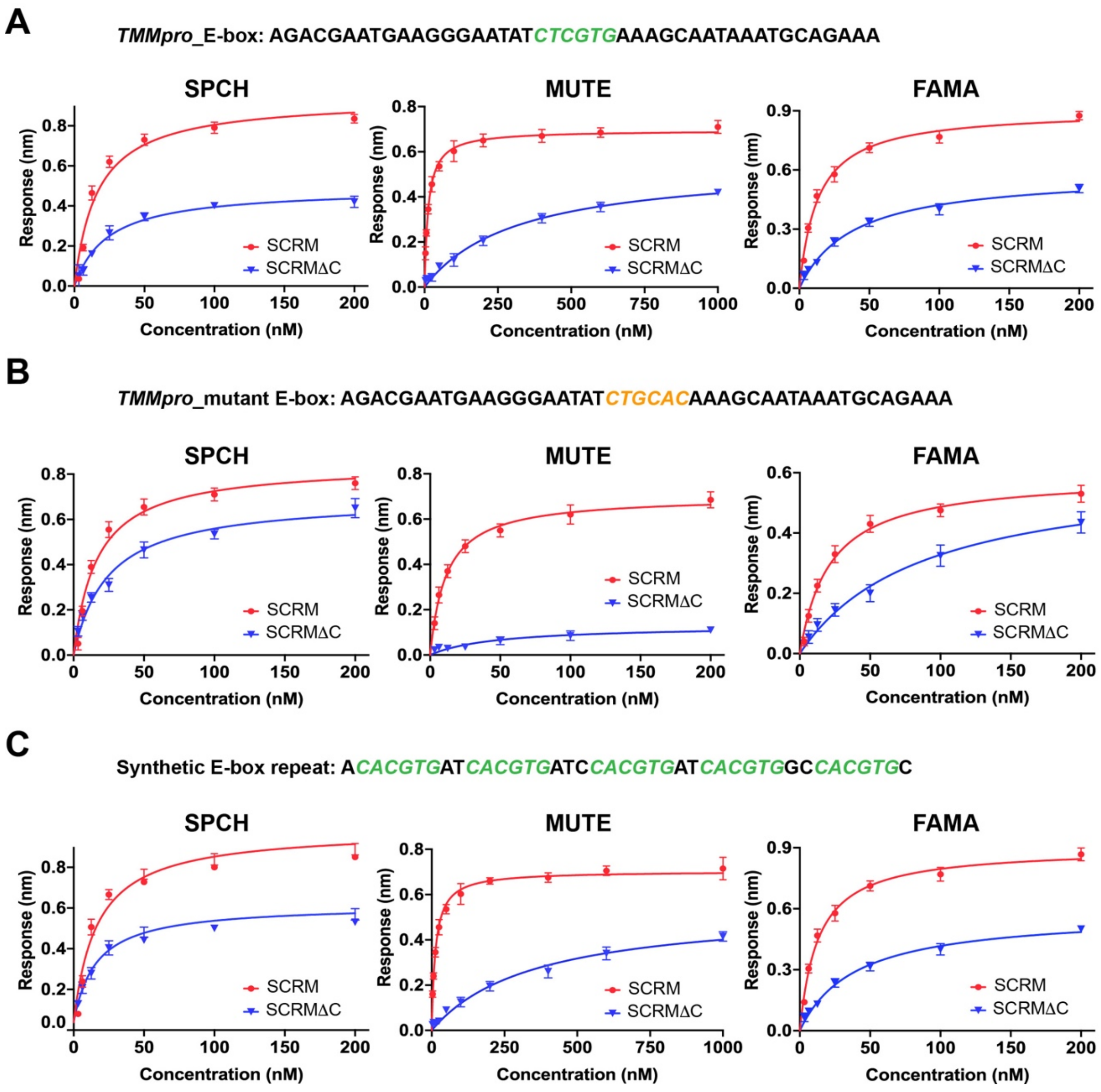

Fig. S5. Quantitative analysis of SCRM/$SCRM_{\Delta C}$ • SPCH/MUTE/FAMA heterodimer interactions in presence of DNA fragments by BLI.

(**A**) *in vitro* binding response curves for *TMM* promoter fragment containing E-box incubated wth SPCH (left), MUTE (middle) and FAMA (right) with GST fused SCRM (red) and $SCRM_{\Delta C}$ (blue). Top, oligo DNA sequence used in assays. E-box is highlighted in green.

(**B**) *in vitro* binding response curves for *TMM* promoter fragment containing mutant E-box (orange) incubated with SPCH (left), MUTE (middle) and FAMA (right) with GST fused SCRM (red) and $SCRM_{\Delta C}$ (blue). Top, oligo DNA sequence used in assays. mutant E-box is highlighted in green, with mutated residues highlighted in red.

(**C**) *in vitro* binding response curves for Synthetic E-box repeat fragment incubated with SPCH (left), MUTE (middle) and FAMA (right) with GST fused SCRM (red) and $SCRM_{\Delta C}$ (blue). Top, oligo DNA sequence used in assays. E-boxes are highlighted in green.

For all assays, SPCH, MUTE and FAMA proteins at seven different concentrations (200, 100, 50, 25, 12.5, 6.25 and 3.125 nM) or (1000, 600, 400, 200, 100, 50, and 25 nM) are subjected to analysis. Data are mean ± s.d., representative of two independent experiments.

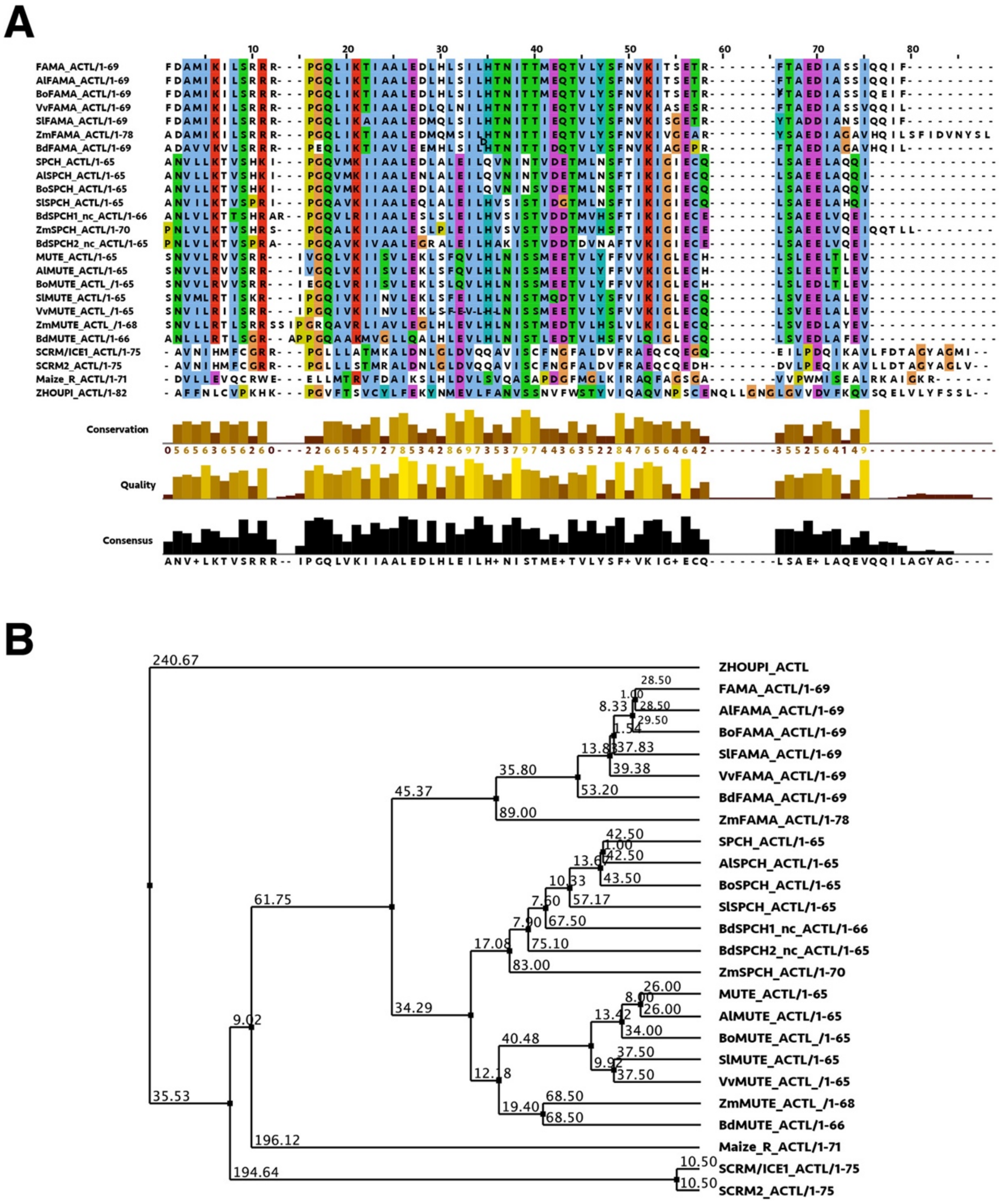

Figure S6. SPCH/MUTE/FAMA orthologs possess highly-conserved C-terminal ACTL domains

(A) Sequence alignment of the ACTL domain from SPCH, MUTE, and FAMA and their orthologs using CLUSTALW and JALVIEW. The ACTL domain from SCRM, SCRM2, ZHOUPI, and Maize R are also included as an outgroup.

(B) The molecular phylogenetic analysis of the ACTL domains from SPCH, MUTE, and FAMA ACTL domains along with SCRM, SCRM2, ZHOUPI, and Maize R. The molecular phylogenetic tree was constructed by using UPGMA (Unweighted Pair Group Method with Arithmetic Mean). The pairwise distances used to cluster the sequences are noted as the percentage mismatch between two sequences at each node.

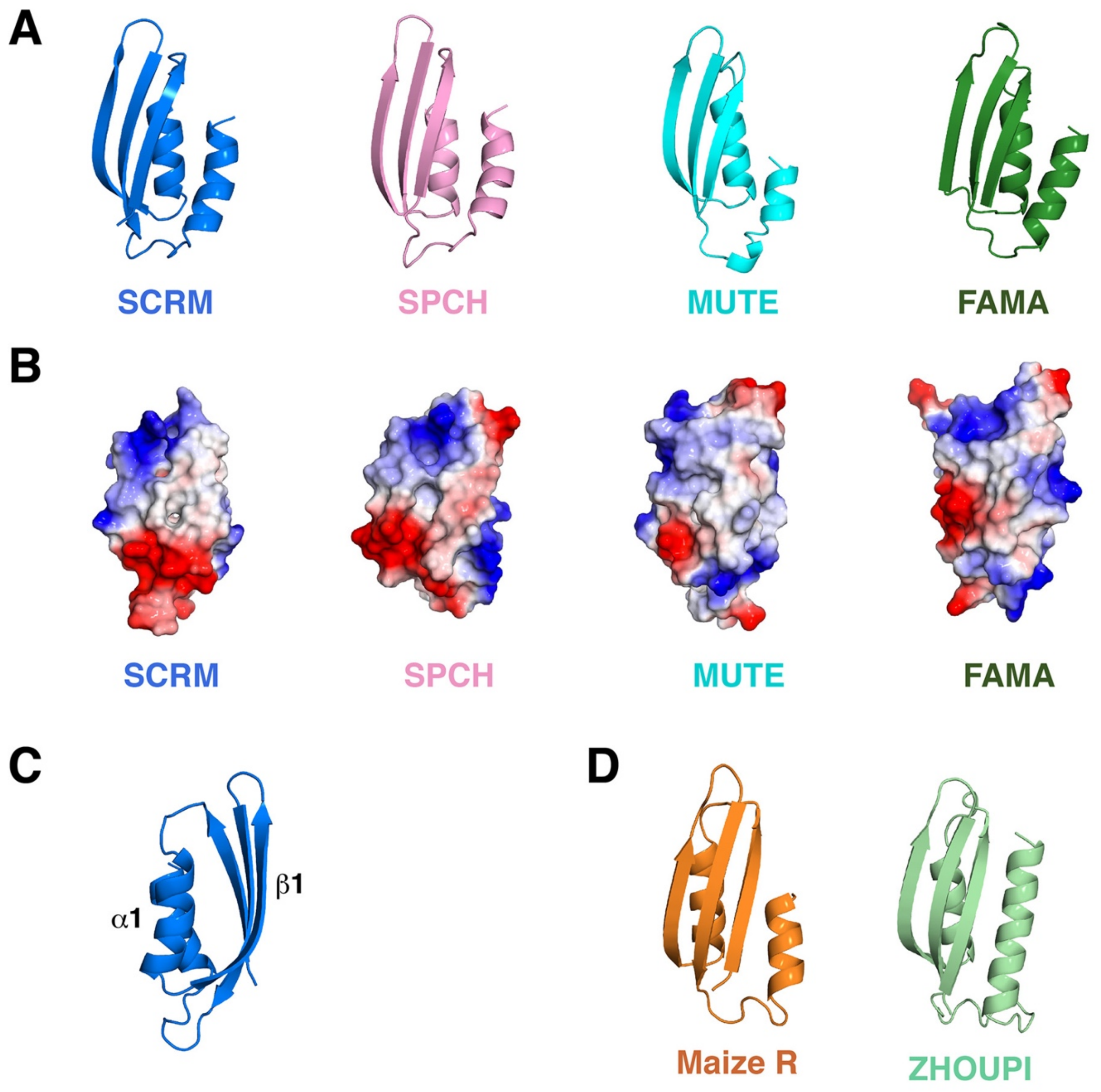

Fig. S7. Modelled ACTL domain structures and surface charge maps
(A) Ribbon representation of the ACTL domain models of SCRM, SPCH, MUTE, and FAMA proteins. See Methods for details in structural modeling.
(B) Electrostatic surface potential map of the ACTL domains of SCRM, SPCH, MUTE, and FAMA proteins. Blue indicates positively charged regions, whereas red shows negatively charged regions. All the molecules are in the same orientation.
(C) A ribbon diagram of SCRM ACTL as an example showing the location of α1 helix and β1 strand that are predicted to serve as intermolecular interaction interface shown in (**B**).
(D) Ribbon representation of ACTL domains of bHLH family Maize R and ZHOUPI proteins.

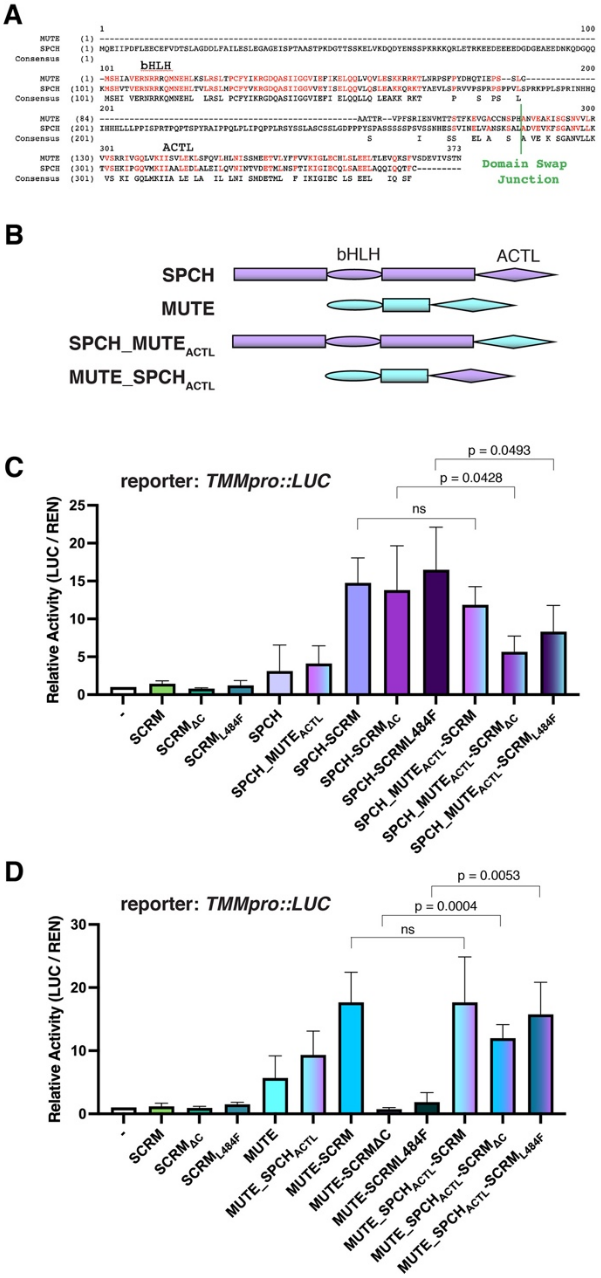

Fig. S8. Domain-swap experiments show that the SPCH ACTL domain is sufficient to induce target gene expression *in planta* with SCRM ACTL mutants

(A) Amino-acid sequence alignment of SPCH and MUTE, indicating the domain-swap junction (green line).

(B) Schematic diagrams of SPCH (lilac), MUTE (cyan), and their complementary domain-swap constructs. $SPCH\_MUTE_{ACTL}$, SPCH backbone with MUTE ACTL domain; $MUTE\_SPCH_{ACTL}$, MUTE backbone with SPCH ACTL domain.

(C and D) Dual-luciferase assays of *TMM* promoter with individual or pairwise combinations of effector SPCH and $SPCH\_MUTE_{ACTL}$ (**C**) as well as MUTE and $MUTE\_SPCH_{ACTL}$ **(D)** with SCRM, $SCRM_{\Delta C}$, and $SCRM_{L484F}$. Relative luciferase activity (LUC/REN) was normalized against the empty vector control. One-tailed Student's t-test was performed for selected pairwise combinations. 2 biological replicates were performed, each with 3 technical replicates. Error bars = s.e.m.

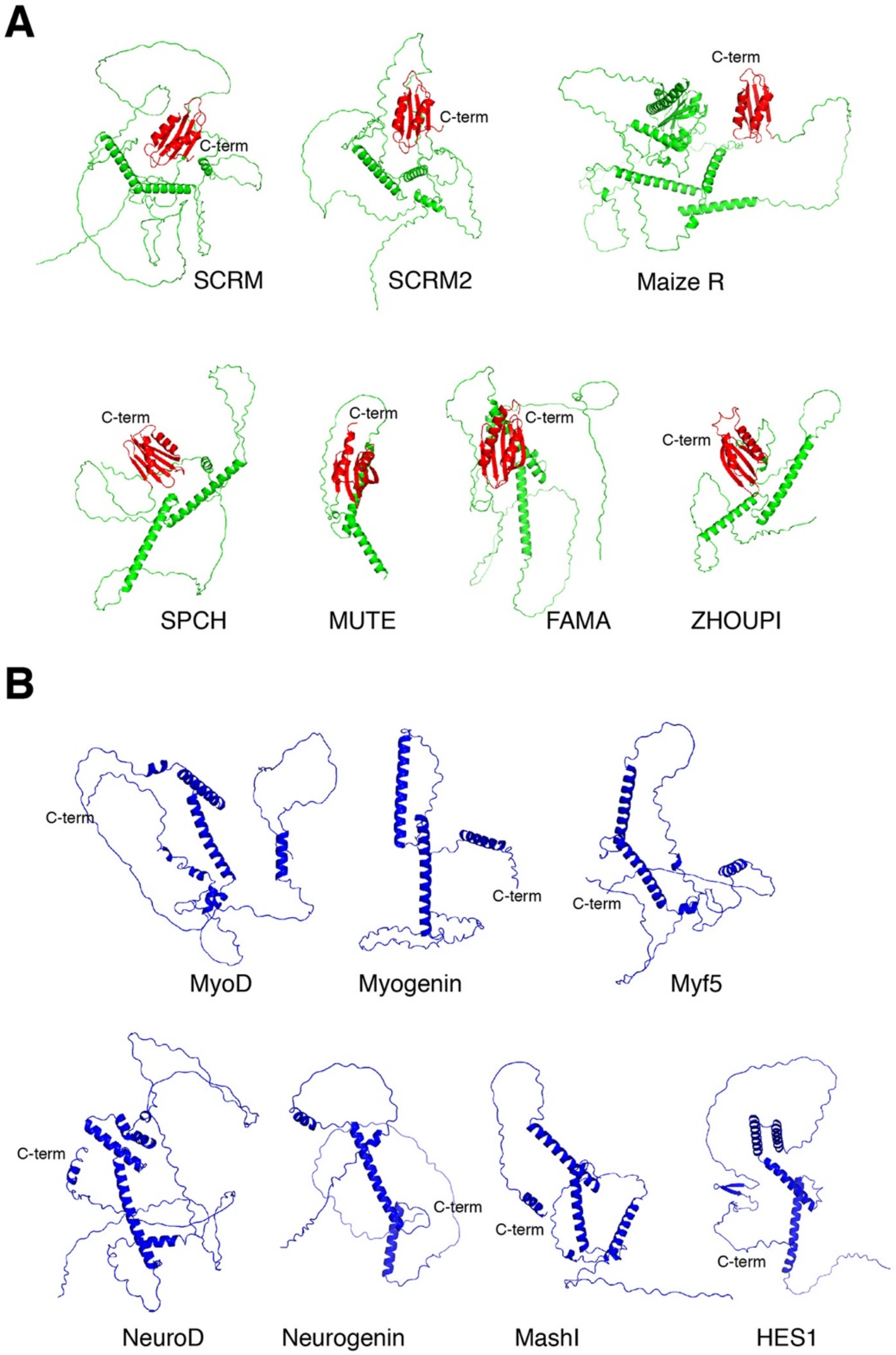

Fig. S9. Unique structural feature of plant developmental bHLH proteins
Shown are predicted full-length structure of plant (**A**) and human (**B**) lineage-specific/developmental regulatory bHLH proteins retrieved from Alphafold Protein Structure Database (https://www.alphafold.ebi.ac.uk/).
(**A**) Plant bHLH proteins (in green) with C-terminal ACTL domain (in red).

(**B**) Human bHLH proteins (in blue) regulating myogenesis (MyoD, Myogenin, Myf5), neurogenesis (NeuroD, Neurogenin, MashI) and a transcriptional repressor HES1. The locations of the C-terminal end are indicated.

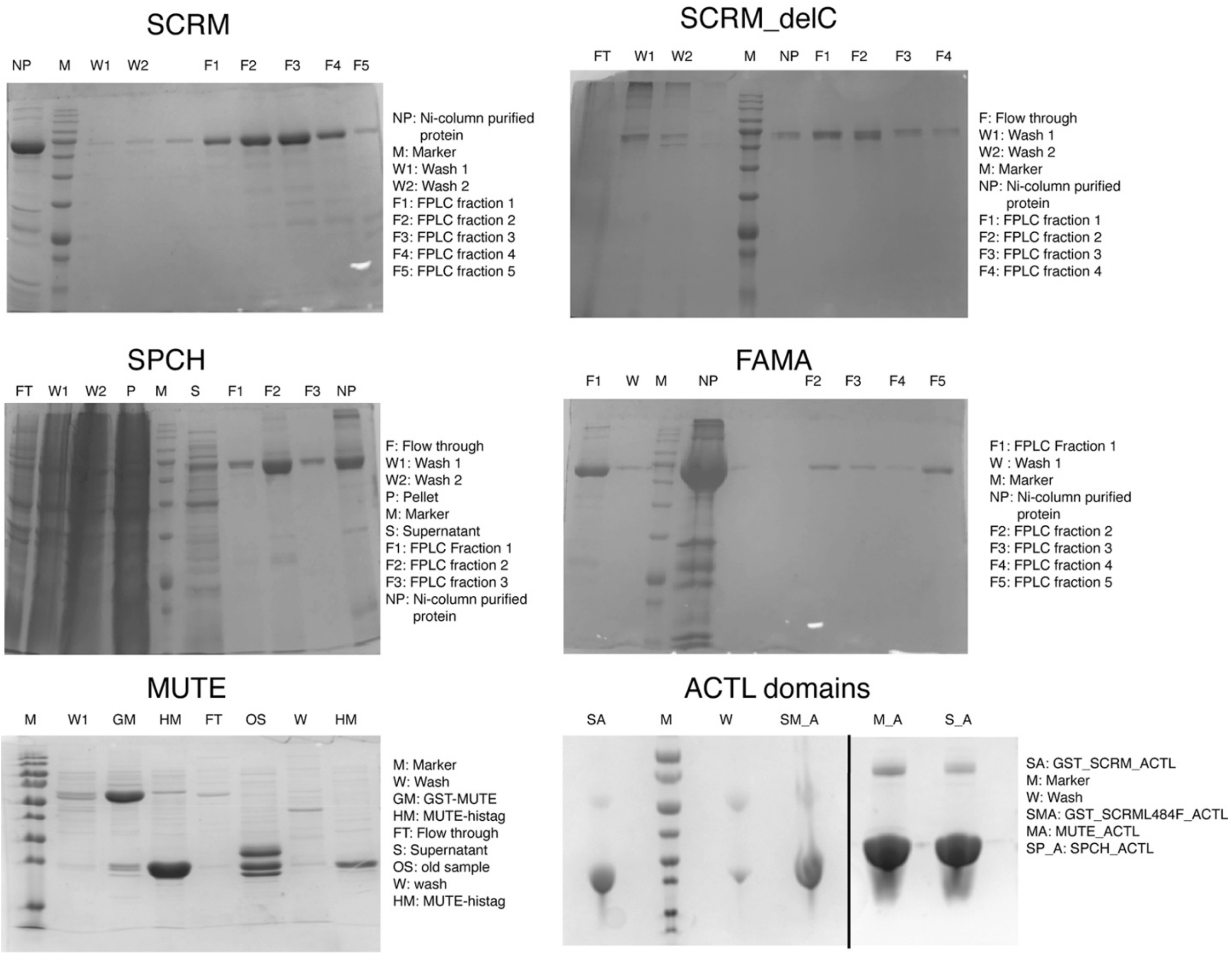

Fig. S10. SDS-PAGE gel images of purified recombinant proteins used for biophysical assays

SUPPLEMENTARY TABLES

Table S1. Thermodynamic parameters for the binding of SCRM / SCRM$_{\Delta C}$ to SPCH and MUTE and FAMA.

The thermodynamic parameters for the binding of SCRM / SCRM$_{\Delta C}$ to SPCH, MUTE and FAMA were measured by ITC in PBS buffer, pH 7.2 buffer at 25 °C. The values reported are the means of two experiments.

	Kd	n	ΔH	-TΔS	ΔG
SCRM-SPCH	6.5 ± 1.6 nM	1.02	-160.3 ± 0.5	152.2 ± 0.4	-7.7 ± 0.2
SCRM_MUTE	10.5 ± 2.1 nM	1.03	-80.5 ± 0.6	73.2 ± 0.6	-7.3 ± 0.2
SCRM$_{\Delta C}$-SPCH	61.1 ± 4.2 nM	0.95	-110.3 ± 0.4	104.2 ± 0.4	-6.0 ± 0.3
SCRM-FAMA	9.6 ± 1.4 nM	0.91	-100.5 ± 0.5	92.7 ± 0.5	-7.3 ± 0.3
SCRM$_{\Delta C}$-FAMA	68.1 ± 5.2 nM	0.96	-95.0 ± 0.6	87.3 ± 0.6	-7.7 ± 0.2

Table S2. Kinetic- and binding constants for SCRM and SCRMΔC to SPCH, MUTE and FAMA using BLI

	k_{on} ($M^{-1}s^{-1}$)	k_{off} (s^{-1})	K_D (nM)	K_D (SS) (nM)
**SCRM**				
SPCH	1.92×10^4	1.11×10^{-4}	5.8 ± 0.5	5.2 ± 1.1
SPCH + E-box (TMM)	3.92×10^4	1.53×10^{-4}	3.9 ± 0.3	4.9 ± 1.4
SPCH + mutE-box (TMM)	2.12×10^4	1.44×10^{-4}	6.8 ± 0.5	5.9 ± 1.3
SPCH + E-box (synthetic)	4.92×10^4	3.54×10^{-4}	7.2 ± 0.4	6.4 ± 1.2
MUTE	2.42×10^4	1.47×10^{-4}	6.1 ± 0.5	7.3 ± 1.5
MUTE + E-box (TMM)	3.92×10^4	2.86×10^{-4}	7.3 ± 0.2	9.3 ± 1.4
MUTE + mutE-box (TMM)	2.08×10^4	2.30×10^{-4}	11.1 ± 0.8	12.2 ± 1.4
MUTE + E-box (synthetic)	5.62×10^4	2.85×10^{-4}	5.1 ± 0.3	6.8 ± 1.2
FAMA	2.22×10^4	1.59×10^{-4}	7.2 ± 0.5	8.2 ± 2.1
FAMA + E-box (TMM)	4.32×10^4	3.95×10^{-4}	9.2 ± 0.3	11.2 ± 2.1
FAMA + mutE-box (TMM)	1.82×10^4	2.82×10^{-4}	15.5 ± 0.6	14.5 ± 1.6
FAMA + E-box (synthetic)	4.32×10^4	3.67×10^{-4}	8.5 ± 0.7	9.2 ± 1.8
**SCRMΔC**				
SPCH	2.05×10^3	1.20×10^{-4}	59.1 ± 1.3	68.2 ± 5.5
SPCH + E-box (TMM)	4.25×10^3	2.08×10^{-4}	49.0 ± 1.3	55.1 ± 4.6
SPCH + mutE-box (TMM)	2.35×10^3	1.83×10^{-4}	78.2 ± 1.2	76.0 ± 6.8
SPCH + E-box (synthetic)	4.85×10^3	1.84×10^{-4}	38.1 ± 1.9	45.2 ± 3.8
MUTE	ND	ND	ND	ND
MUTE + E-box (TMM)	2.85×10^3	1.60×10^{-3}	565.2 ± 3.5	595.2 ± 13.8
MUTE + mutE-box (TMM)	ND	ND	ND	ND
MUTE + E-box (synthetic)	3.10×10^3	1.70×10^{-3}	545.3 ± 5.5	568.0 ± 15.8
FAMA	2.91×10^3	1.60×10^{-4}	55.1 ± 1.3	52.1 ± 3.6
FAMA + E-box (TMM)	3.91×10^3	2.63×10^{-4}	68.3 ± 1.4	74.2 ± 4.6
FAMA + mutE-box (TMM)	2.61×10^3	2.21×10^{-4}	85.0 ± 1.8	93.2 ± 4.8
FAMA + E-box (synthetic)	3.91×10^3	2.30×10^{-4}	59.1 ± 1.3	62.2 ± 5.6

Table S3. Thermodynamic parameters for the binding of the SCRM / $SCRM_{L484F}$ ACTL domains to SPCH and MUTE ACTL domains

The thermodynamic parameters for the binding of SCRM / $SCRM_{L484F}$ to SPCH and MUTE were measured by ITC in PBS buffer, pH 7.2 buffer at 25 °C. The values reported are the means of two experiments.

	**Kd**	**n**	**ΔH**	**-TΔS**	**ΔG**
SCRM-SPCH	1.2 ± 0.6 μM	0.95	-9.2 ± 0.2	1.1 ± 0.2	-8.0 ± 0.1
$SCRM_{L484F}$-SPCH	1.7 ± 0.8 μM	0.85	-9.6 ± 0.2	1.5 ± 0.2	-8.0 ± 0.1
SCRM-MUTE	5.1 ± 1.2 μM	0.95	-15.6 ± 0.2	2.5 ± 0.3	-13.1 ± 0.2
